# Inter-hospital ICU-to-ICU transfer of critically ill COVID-19 patients is not associated with increased mortality: a systematic review with exploratory meta-analysis

**DOI:** 10.1038/s41598-026-62965-7

**Published:** 2026-08-08

**Authors:** Jonas Brock, Harald Lux, Michael Bauer, Petra Dickmann

**Affiliations:** 1 Department of Anaesthesiology and Intensive Care Medicine, Jena University Hospital, Jena, Germany; 2 Leibniz Centre for Photonics in Infection Research (LPI), Jena, Germany; 3 Thuringian Centre for Quality and Research in Emergency Medicine and Emergency Medical Services (ThuZenQ), Jena University Hospital, Jena, Germany

**Keywords:** COVID-19, Inter-hospital transfer, Patient transfer, Intensive care unit, Critically ill, Mortality, Length of stay, Mechanical ventilation, Systematic review, Meta-analysis, Diseases, Health care, Medical research, Risk factors

## Abstract

During the COVID-19 pandemic, inter-hospital transfer of critically ill patients between intensive care units was widely used to manage capacity shortages. While several individual studies have compared outcomes of transferred and non-transferred patients, no systematic synthesis of this evidence exists. This systematic review aims to determine whether inter-hospital ICU-to-ICU transfer of adult COVID-19 patients is associated with increased mortality or other adverse clinical outcomes. We systematically searched PubMed, Web of Science, and Scopus for observational studies comparing clinical outcomes of adult COVID-19 patients who underwent inter-hospital ICU-to-ICU transfer with non-transferred ICU patients. Two reviewers screened, selected, and extracted data independently and in duplicate. Risk of bias was assessed using the Newcastle-Ottawa Scale and ROBINS-I, and certainty of evidence using GRADE. Exploratory random-effects meta-analyses were performed separately for each effect measure, with Hartung-Knapp and crude-effect sensitivity analyses. Nine observational studies from seven countries were included (approximately 6,100 transferred and 29,200 non-transferred ICU patients). None of the adjusted effect estimates showed a statistically significant mortality disadvantage for transferred patients. Pooled adjusted subgroup estimates were an odds ratio of 1.29 (95% CI 0.84 to 1.98; k = 3) and a hazard ratio of 0.71 (95% CI 0.34 to 1.46; k = 2). Length of stay was longer in transferred patients in most studies. The available observational evidence did not show a clear increase in mortality among transferred compared with non-transferred COVID-19 ICU patients. These findings support the use of inter-hospital transfer as a safe strategy for managing ICU surge capacity. PROSPERO CRD420261356915.

**Supplementary Information:**

The online version contains supplementary material available at 10.1038/s41598-026-62965-7.

## Introduction

The COVID-19 pandemic caused by SARS-CoV-2 put pressure on intensive care unit (ICU) capacity worldwide. To address these constraints, many countries adopted inter-hospital transfer of critically ill patients between ICUs as a strategy to redistribute the burden and maintain access to critical care^1,2^.

Inter-hospital ICU transfers are typically motivated by two factors: capacity constraints at overwhelmed facilities, and the need for specialized treatments such as extracorporeal membrane oxygenation (ECMO)^3^. During the pandemic, the majority of transfers were capacity-driven, making this a system-level strategy rather than an individual clinical decision.

At the individual patient level, inter-hospital transfer exposes critically ill patients to physiological stress, including hemodynamic changes, risk of infection, and interruption of ongoing care during transport^4,5^. Pre-pandemic data from general ICU populations suggested that inter-hospital transfer may be associated with longer ICU stays and higher mortality, although these associations were generally attenuated or absent after adjustment for illness severity^6,7^.

Since the onset of the pandemic, several observational studies from different countries have compared clinical outcomes of transferred and non-transferred COVID-19 ICU patients. These studies vary considerably in their effect measures (odds ratios, hazard ratios, risk differences), mortality timepoints, and approaches to methodological challenges such as immortal time bias. Despite this growing body of individual evidence, no systematic review has synthesized the available data specifically for inter-hospital ICU-to-ICU transfer with a non-transferred comparator.

This systematic review aims to close this gap by providing a comprehensive synthesis of the existing evidence on inter-hospital ICU-to-ICU transfer of critically ill COVID-19 patients. The primary objective was to determine whether inter-hospital ICU-to-ICU transfer is associated with increased mortality. Secondary objectives included the comparison of ICU length of stay, hospital length of stay, and duration of mechanical ventilation between transferred and non-transferred patients.

## Methods

### Protocol and registration

This systematic review was conducted according to the PRISMA 2020 guidelines^8^ and was registered with PROSPERO (CRD420261356915) prior to completion of literature screening.

Two analyses were added post hoc and are reported as exploratory deviations from the registered protocol: a crude common-measure (odds ratio) sensitivity meta-analysis and a ROBINS-I risk-of-bias assessment.

### Eligibility criteria

Eligibility criteria were defined based on the PICO framework. We defined the exposure of interest as inter-hospital ICU-to-ICU transfer, i.e. the movement of a critically ill patient between the intensive care units of two different hospitals. Terms used interchangeably in the primary studies, including inter-hospital transfer, interhospital transfer, transport, retrieval, and evacuation, were treated as referring to this same exposure, whereas ‘transport’ is used for the journey itself. We included observational studies (cohort, matched cohort, case-control, or registry-based) with a comparative design that reported clinical outcomes of adult (≥ 18 years) COVID-19 patients who underwent inter-hospital ICU-to-ICU transfer compared with non-transferred ICU patients. A non-transferred comparator group was required for inclusion. The primary outcome was mortality at any timepoint; secondary outcomes were ICU length of stay, hospital length of stay, and duration of mechanical ventilation.

Studies were excluded if they lacked a non-transferred comparator group, reported only intra-hospital transfers, examined exclusively ECMO-specific transfer programs without a general ICU comparator, focused on transport-related adverse events without post-transfer clinical outcomes, or were not original research (e.g., editorials, reviews, letters without original data).

### Search strategy

PubMed, Web of Science, and Scopus databases were searched electronically on 15th February 2026. The search strategy combined three conceptual blocks using Boolean operators (Table 1). Terms within each block were linked with OR; blocks were combined with AND. Reference lists of included studies were checked for additional relevant publications.

**Table 1:** Search strategy applied across PubMed, Web of Science, and Scopus (search date: 15 February 2026). Database-specific syntax was adapted per database; full strategies are provided in Additional file 1.

Component	Search terms
COVID-19	COVID-19 OR SARS-CoV-2 OR coronavirus disease 2019 OR 2019-nCoV OR coronavirus
ICU/Critical Care	intensive care OR critical care OR critically ill OR ICU OR intensive care unit
Inter-hospital Transfer	patient transfer OR transfer OR transport OR evacuation OR repatriation OR interhospital OR inter-hospital OR interfacility OR inter-facility OR retrieval
Combined	Component 1 AND Component 2 AND Component 3

### Study selection

Study selection followed a two-stage process. First, titles and abstracts of all unique records were screened against the predefined eligibility criteria. Studies classified as potentially eligible or uncertain at this stage were advanced to full-text review. At the full-text stage, each study was assessed against the complete set of inclusion and exclusion criteria. During data extraction, studies that did not meet the PICO criteria defined in this study were excluded with documented reasons.

Titles and abstracts of all unique records were screened independently by two reviewers (JB and HL). Any record judged potentially eligible or uncertain by either reviewer was advanced to full-text assessment. Full-text eligibility assessment and data extraction were both performed independently and in duplicate by the same two reviewers, and disagreements at each stage were resolved by discussion until consensus was reached. The screening was repeated before manuscript preparation to capture any studies published during the review; no additional eligible studies were identified.

### Data extraction

Data were extracted into a standardized form with 70 fields covering study characteristics, population demographics, transfer details, clinical outcomes, and effect measures. Adjusted effect estimates were preferred over unadjusted estimates where available. The complete extraction table is provided in Additional file 3.

### Risk of bias assessment

Risk of bias was assessed using the Newcastle-Ottawa Scale (NOS) for cohort studies. Each study was rated on the three NOS domains (Selection, Comparability, Outcome) and categorised as low (7 to 9 of 9 stars), moderate (4 to 6), or high (0 to 3) risk of bias; for the Comparability domain the pre-specified confounders were age and disease severity, and the presence of methods to address immortal time bias was assessed separately. To complement the NOS, which is limited in its sensitivity to time-varying exposure and confounding by indication, we additionally assessed risk of bias for the primary outcome (mortality) using the ROBINS-I tool across its seven domains, with explicit consideration of confounding, selection of participants (immortal time bias), and classification of the transfer exposure (Additional file 7). Assessments were made independently by three assessors; for the ROBINS-I assessment a large language model served as one of the three assessors, with all judgments verified by a human author (see Declarations).

### Certainty of evidence

Certainty of evidence was assessed using the GRADE approach^9^ for all four pre-specified outcomes. Detailed results are presented in Additional file 4.

### Data synthesis and meta-analysis

All included studies were synthesized narratively. An exploratory meta-analysis was performed for studies reporting compatible ratio-based effect measures (odds ratios or hazard ratios) for mortality. Studies reporting other measures (risk differences, incidence rate ratios, relative risk ranges) or no adjusted measure were included only in the narrative synthesis.

The generic inverse-variance method with DerSimonian-Laird random-effects models was used for pooling. Odds ratios and hazard ratios were analysed in separate subgroups, and no overall pooled estimate was calculated across subgroups because combining different effect measures is not recommended^10^. One study (Stark 2023^16^ reported an odds ratio for survival (1.14, 95% CI 0.43 to 3.10); we inverted it to a mortality odds ratio by taking the reciprocal of the point estimate and of each confidence limit (1/1.14 = 0.88; 95% CI 1/3.10 to 1/0.43, i.e. 0.32 to 2.33), so that all estimates represent mortality.

As a sensitivity analysis addressing the differing effect measures, we additionally pooled crude odds ratios derived from 2 × 2 mortality counts extracted from each report, using both DerSimonian-Laird and Hartung-Knapp-Sidik-Jonkman random-effects methods, and reported τ², I², and a 95% prediction interval (Additional file 9). Prespecified sensitivity analyses excluded the study with a day-5 conditioning design (Grimaud 2026^13^, studies that did not address immortal time bias, and the potentially overlapping French cohorts. Because odds ratios and hazard ratios are not combinable and mortality timepoints differed, all pooled estimates are exploratory.

## Results

### Study selection

**Fig. 1:**
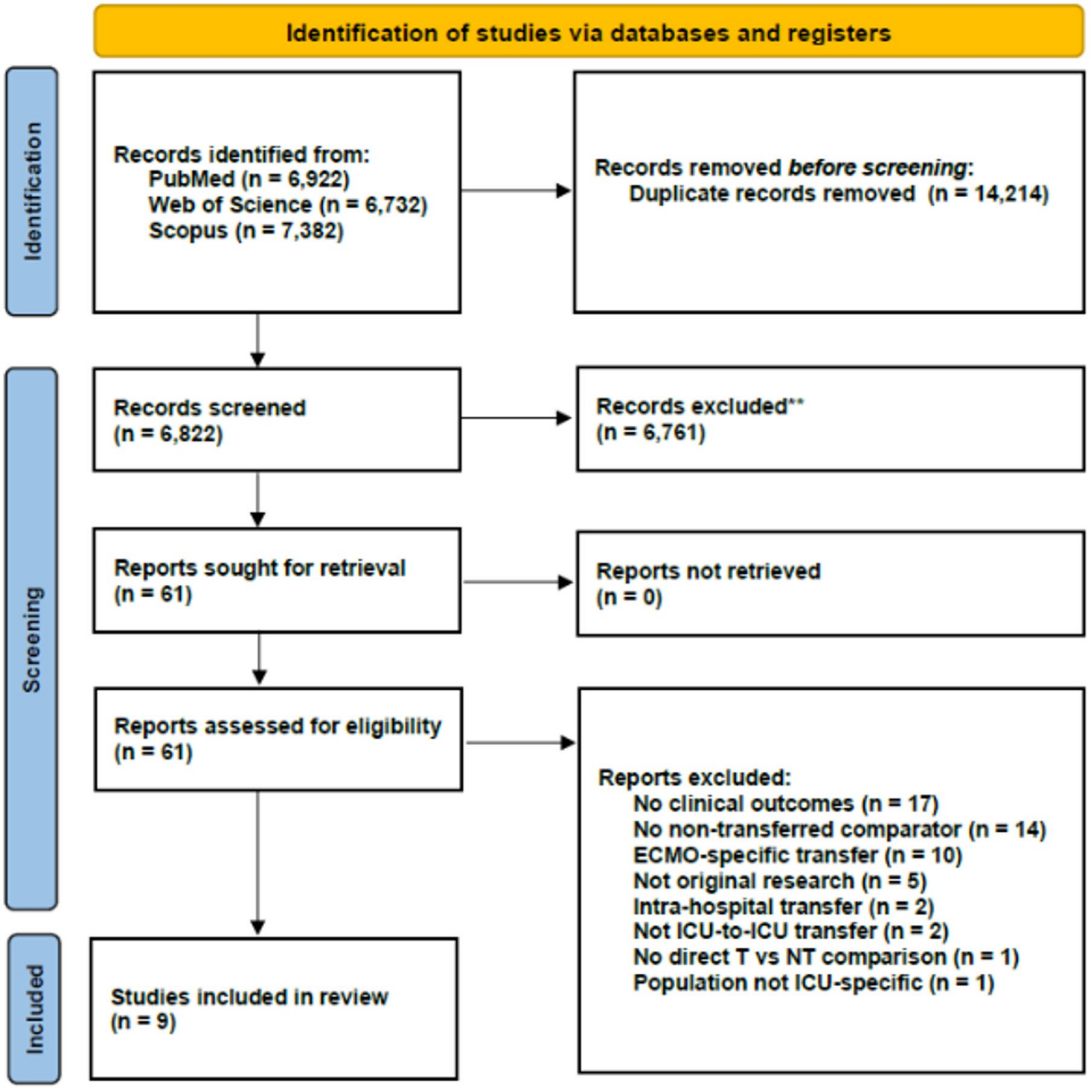
PRISMA 2020 flow diagram. Records identified from PubMed (*n* = 6,922), Web of Science (*n* = 6,732), and Scopus (*n* = 7,382). After duplicate removal (*n* = 14,214), 6,822 records were screened. Forty-eight were excluded at full text, four during data extraction. Nine studies were included in the synthesis, five in the exploratory meta-analysis.

The database search identified 21,036 records (PubMed: 6,922; Web of Science: 6,732; Scopus: 7,382). After removal of 14,214 duplicates, 6,822 unique records remained for title and abstract screening. Of these, 6,761 were excluded, leaving 61 studies for full-text assessment (Fig. 1).

At the full-text stage, 52 reports were excluded: no comparative clinical outcomes (*n* = 17), no non-transferred comparator (*n* = 14), ECMO-specific transfer programmes without a general ICU comparator (*n* = 10), not original research (*n* = 5), intra-hospital transfer only (*n* = 2), not ICU-to-ICU transfer (*n* = 2), no direct transfer-versus-non-transfer comparison (*n* = 1), and a population that was not ICU-specific (*n* = 1) (Additional file 2).

Nine studies met all eligibility criteria and were included in the qualitative synthesis, five of which contributed to the exploratory meta-analysis (Fig. 1). Four of these exclusions (two lacking a non-transferred comparator, one without a direct transfer-versus-non-transfer comparison, and one with a non-ICU-specific population) were identified only after detailed full-text data assessment and are reported as full-text exclusions accordingly.

### Study characteristics

The nine included studies^11–19^ were published between 2021 and 2026 and originated from seven countries: France (*n* = 3), and one each from Australia, Germany, the Netherlands, Sweden, the United Kingdom, and the United States (Table 2). Eight were retrospective and one prospective cohort studies. Settings ranged from single-center to nationwide registries (1 to 111 centers). Study periods spanned March 2020 to December 2021, covering pandemic waves one through four.

The total population comprised approximately 6,100 transferred and 29,200 non-transferred ICU patients. However, the three French first-wave cohorts (Sanchez 2021^15^, Grimaud 2026^13^, Painvin 2021^14^ overlap in time, region, and data sources: the national Sanchez cohort very likely subsumes the transferred patients described in more detail by Grimaud and Painvin. The number of unique patients is therefore lower than the arithmetic sum, and these totals should not be interpreted as counts of unique individuals (Additional file 8). Sample sizes ranged from 108 (Stark 2023^16^ to 17,531 (Sanchez 2021^15^).

**Table 2:** Characteristics of included studies. T = transferred; NT = non-transferred; NR = not reported. Setting shows number of centers in parentheses. Studies are ordered alphabetically by first author.

Study	Country	Design	Setting/Centers	Study Period	*n* (T/NT)
Chen (2021)^11^	USA	Retrospective cohort	Single center (1)	2020-03-17 to 2020-10-14	117/181
Cini (2023)^12^	Australia	Retrospective cohort	Multi center (63)	2020-01-01 to 2022-04-01	328/4,879
Grimaud (2026)^12^	France	Matched cohort	Multi center (111)	2020-03-13 to 2020-04-10	285/667
Painvin (2021)^14^	France	Retrospective cohort	Multi center (3)	2020-03-15 to 2020-04-15	68/65
Sanchez (2021)^15^	France	Prospective	Nationwide	2020-03-01 to 2020-06-21	2,228/15,303
Stark (2023)^16^	UK	Retrospective cohort	Single center (1)	2020-10-01 to 2021-03-31	30/78
Suski (2024)^17^	Germany	Retrospective cohort	Multi center (4)	2021-08-01 to 2021-12-31	115/100
Toss Agegard (2025)^18^	Sweden	Retrospective cohort	Single center (1)	2020-03-01 to 2021-06-30	133/532
Wortel (2022)^19^	Netherlands	Retrospective cohort	Nationwide (73)	2020-03-01 to 2021-07-01	2,814/7,395

### Patient demographics

Patient demographics are summarised in Table 3. Median age ranged from 53 to 65 years in transferred and 57 to 69 years in non-transferred patients, and the proportion of males was higher in both groups (57 to 77%). Where reported, disease severity was measured with different tools (APACHE II/III/IV, SAPS II, or SOFA) and was broadly similar between groups, although several studies did not report a severity score, limiting comparison. The proportion of patients receiving invasive mechanical ventilation — variably defined across studies as ventilation at first ICU admission, at transfer, or at receiving-ICU admission — was higher in transferred patients in several cohorts (e.g., 98.5% vs. 40.0% in Toss Agegard 2025^18^; 83.7% vs. 56.2% in Wortel 2022^19^.

**Table 3:** Patient demographics and baseline severity. Each cell shows transferred (T, top) and non-transferred (NT, bottom) values. NR = not reported; MV = mechanically ventilated at ICU admission. Age: median (IQR) unless noted as mean (± SD). Severity: APACHE II/III/IV, SAPS II, or SOFA as reported.

Study	Age median (IQR)	Male %	BMI	Severity Score (Type)	MV %	Diabetes %
Chen (2021)^11^	**T** 57 (45–64) **NT** 61 (52–69)	**T** 65.8 **NT** 63.0	NR	NR	NR	**T** 50.4 **NT** 54.7
Cini (2023)^12^	**T** 53 (45–61) **NT** 60 (46–70)	**T** 68.6 **NT** 62.2	**T** 32.5 **NT** 30.1	**T** 14 **NT** 14 *APACHE II*	**T** 71.2 **NT** 38.1	**T** 29 **NT** 32
Grimaud (2026)^13^	**T** 64 (56–71) **NT** 64 (55–70)	**T** 74.4 **NT** 72.9	NR	**T** 40 **NT** 40 *SAPS II*	**T** 47.0 **NT** 46.6	**T** 29.3 **NT** 31.4
Painvin (2021)^14^	**T** 62 (55–70) **NT** 64 (54–71)	**T** 69 **NT** 74	**T** 29 **NT** 29	**T** 33 **NT** 35 *SAPS II*	**T** 100 **NT** 100	NR
Sanchez (2021)^15^	**T** 61.2 (± 12.6) **NT** 63.0 (± 15.5)	**T** 74.3 **NT** 68.4	NR	NR	NR	NR
Stark (2023)^16^	**T** 63 (59.25–69.75) **NT** 57 (48.5–64)	**T** 63.3 **NT** 57.7	NR	**T** 14 **NT** 13 *APACHE II*	NR	**T** 26.7 **NT** 20.5
Suski (2024)^17^	**T** 62 (55–73) **NT** 69 (52.5–77)	**T** 71.3 **NT** 60.0	**T** 29.4 **NT** 28.4	**T** 13 **NT** 13 *APACHE II*	**T** 63.0 **NT** 16.0	NR
Toss Agegard (2025)^18^	**T** 65 (57–71) **NT** 64 (55–72)	**T** 77.4 **NT** 70.7	NR	NR	**T** 98.5 **NT** 40.0	NR
Wortel (2022)^19^	**T** 64 (57–71) **NT** 64 (56–72)	**T** 72.2 **NT** 69.2	**T** 28.7 **NT** 28.6	**T** 58 **NT** 60 *APACHE III*	**T** 83.7 **NT** 56.2	**T** 20.9 **NT** 23.5

### Mortality

All nine studies reported mortality, with varying timepoints: hospital mortality (*n* = 5), ICU and hospital mortality (*n* = 1), 28-day (*n* = 1), 30-day (*n* = 1), and 180-day (*n* = 1). Crude mortality rates and adjusted estimates are presented in Table 4.

In the adjusted analyses, transfer was not clearly associated with increased mortality. Eight studies provided adjusted effect estimates, and none showed a statistically significant disadvantage for transferred patients. Among the studies reporting odds ratios, Chen 2021^11^(OR 1.45, 95% CI 0.86 to 2.43), Painvin 2021^14^(OR 1.20, 0.35 to 4.09), and Stark 2023^16^(OR for survival 1.14; inverted for mortality 0.88, 0.32 to 2.33) all had confidence intervals crossing the null. Of the two studies reporting hazard ratios, Wortel 2022^19^ found no association (HR 0.99, 0.91 to 1.08) and Toss Agegard 2025^18^ found lower 30-day mortality in transferred patients (HR 0.47, 0.30 to 0.76). Suski 2024^17^ reported no significant difference (*p* = 0.663) without an adjusted effect estimate.

Among the studies with alternative effect measures, Cini 2023^12^ reported a risk difference of −5.0% points (95% CI −10.0 to −0.03) favoring transfer. Sanchez 2021^15^ reported distance-stratified relative risks ranging from 0.38 to 0.98. Grimaud 2026^13^ reported an incidence rate ratio of 0.14 (0.10 to 0.19), though this estimate was strongly influenced by a day-5 conditioning design that excluded early deaths in non-transferred patients from the analysis.

### Exploratory meta-analysis

**Fig. 2:**
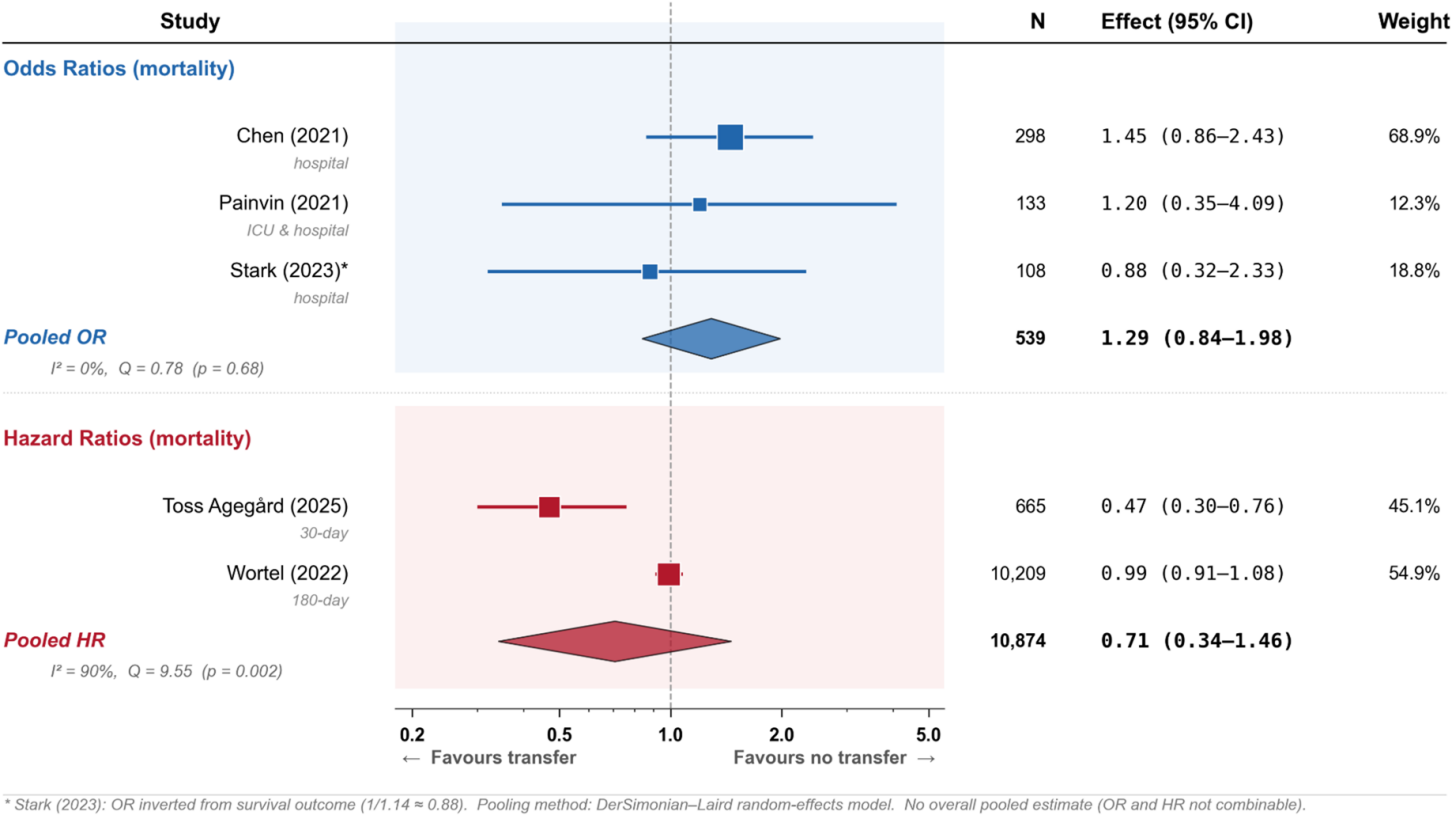
Forest plot of mortality. Exploratory meta-analysis with DerSimonian-Laird random-effects models. OR and HR subgroups shown separately; no overall estimate. Square size proportional to weight. *Stark 2023: OR inverted from survival outcome.

Five studies with compatible ratio-based measures were included in the exploratory meta-analysis (Fig. 2). In the OR subgroup (k = 3: Chen^11^, Painvin^14^, Stark^16^, inverted), the pooled OR was 1.29 (95% CI 0.84 to 1.98; I²=0%; Q = 0.78, *p* = 0.68). In the HR subgroup (k = 2: Wortel^19^, Toss Agegard^18^, the pooled HR was 0.71 (95% CI 0.34 to 1.46; I²=90%; Q = 9.55, *p* = 0.002). The high heterogeneity in the HR subgroup reflects differences in follow-up duration (180-day vs. 30-day) and methods for addressing immortal time bias. No overall estimate was calculated across subgroups because odds ratios and hazard ratios are not combinable^10^.

In a sensitivity analysis using a common effect measure, the pooled crude odds ratio for mortality was 0.90 (95% CI 0.74 to 1.09; k = 8) with the Hartung-Knapp method, with substantial heterogeneity (I²=71%; τ²=0.034) and a 95% prediction interval of 0.54 to 1.49 (Additional file 9). The estimate was stable when excluding studies that did not address immortal time bias (OR 0.86, 95% CI 0.67 to 1.11) and when excluding potentially overlapping French cohorts (OR 0.90, 95% CI 0.73 to 1.12). Including Grimaud 2026, whose day-5 conditioning design excludes early deaths among controls, shifted the estimate downward (OR 0.77) and increased heterogeneity (I²=87%), confirming it as an outlier. Given the small number of studies, the differing mortality timepoints, and the wide prediction interval, these pooled estimates are exploratory and hypothesis-generating.

### Length of stay

**Fig. 3:**
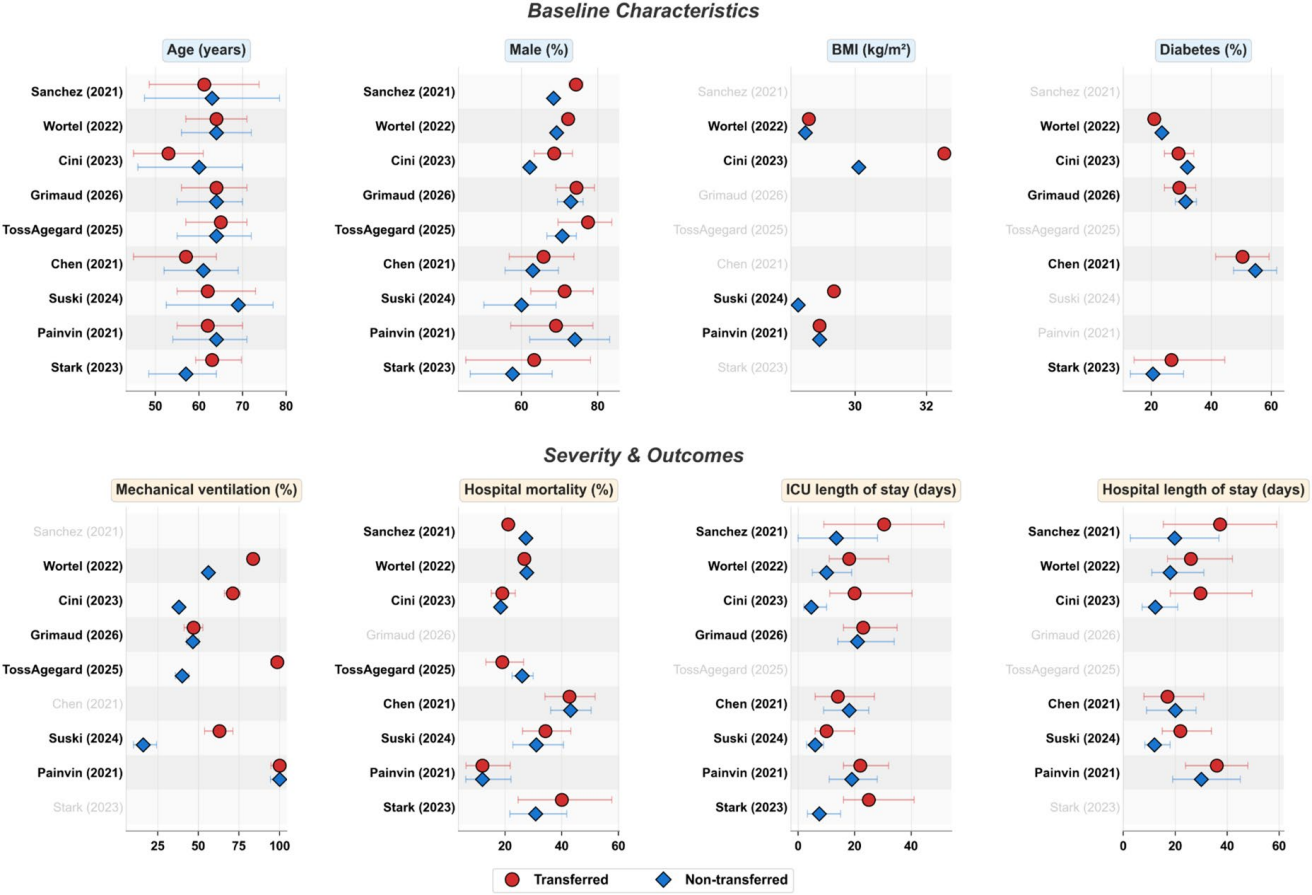
Paired comparison of transferred (orange circles) and non-transferred (blue diamonds) COVID-19 ICU patients across nine studies. Upper panels: age, sex, BMI, diabetes; lower panels: mechanical ventilation, hospital mortality, ICU length of stay, hospital length of stay. Gray labels indicate data not reported. Error bars represent interquartile ranges or 95% Wilson confidence intervals.

In most studies, measures of length of stay were longer in transferred patients (ICU length of stay was reported by eight studies; median 10 to 30.4 vs. 4.6 to 21 days; Table 4; Fig. 3), although Chen 2021^11^ reported a longer stay in the non-transferred group. These comparisons are limited by differing anchor points: registry-based studies (e.g., Cini 2023^12^ capture the total cross-hospital episode, whereas some single-centre studies measure length of stay only from arrival at the receiving ICU (Additional file 8).

**Table 4:** Clinical outcomes and effect estimates. T = transferred; NT = non-transferred; LOS = length of stay (median, days); NR = not reported; OR = odds ratio; HR = hazard ratio; RR = relative risk; IRR = incidence rate ratio; RD = risk difference. Studies ordered alphabetically. ᵃ Stark 2023: OR refers to survival; inverted for mortality = 0.88 (0.32–2.33).

Study	Mortality %	ICU LOS days	Hospital LOS days	Effect Measure	Estimate (95% CI)	*p*
Chen (2021)^11^	T 42.7 NT 43.1	T 14 NT 18	T 17 NT 20	OR	1.45 (0.86–2.43)	NR
Cini (2023)^12^	T 19.0 NT 18.4	T 20 NT 4.6	T 29.7 NT 12.3	RD	−5.0 (− 10 to − 0.03)	NR
Grimaud (2026)^13^	T 7.0 NT 29.8	T 23 NT 21	NR	IRR	0.14 (0.10–0.19)	< 0.0001
Painvin (2021)^14^	T 12 NT 12	T 22 NT 19	T 36 NT 30	OR	1.20 (0.35–4.09)	0.76
Sanchez (2021)^15^	T 21.1 NT 27.3	T 30.4 NT 13.5	T 37.2 NT 19.8	RR	0.38–0.98 (range)	< 0.0001 to 0.82
Stark (2023)ᵃ^16^	T 40.0 NT 30.8	T 25 NT 7.5	NR	OR (surv.)	1.14 (0.43–3.1)	0.80
Suski (2024)^17^	T 34.2 NT 31.0	T 10 NT 6	T 22 NT 12	—	NR	0.663
Toss Agegard (2025)^18^	T 19 NT 26	NR	NR	HR	0.47 (0.30–0.76)	< 0.05
Wortel (2022)^19^	T 26.8 NT 27.6	T 18 NT 10	T 26 NT 18	HR	0.99 (0.91–1.08)	NR

Hospital length of stay (six studies) showed a similar pattern. Duration of mechanical ventilation was reported by three studies with mixed results.

### Risk of bias

On the Newcastle-Ottawa Scale, risk of bias was low in seven studies (7 to 9 of 9 stars) and moderate in two (Suski 2024^17^: 6/9; Painvin 2021^14^: 5/9), with no study rated high risk; the weakest domain was Comparability, as four of nine studies did not adjust for disease severity (Additional file 5). All studies received maximum stars on the Outcome domain because mortality was ascertained from complete hospital or registry records with follow-up adequate for the reported endpoint; this domain reflects the method and completeness of outcome ascertainment rather than standardisation of the timepoint, which differed across studies. Because the Newcastle-Ottawa Scale incompletely captures time-varying exposure and confounding by indication, we also applied ROBINS-I to the primary outcome: risk of bias was serious in eight of nine studies and moderate in one (Wortel 2022^19^, driven chiefly by confounding by indication (serious in eight studies) and by selection effects related to immortal time bias (Additional file 7). This more conservative rating is consistent with the very low GRADE certainty.

Immortal time bias was fully addressed in three studies (Wortel 2022^19^: left truncation; Grimaud 2026^13^: day-5 conditioning; Toss Agegard 2025^18^: landmark sensitivity analysis), partially addressed in three (Chen^11^, Cini^12^, Sanchez^15^, and not addressed in three (Painvin^14^, Stark^16^, Suski^17^.

### Certainty of evidence

GRADE certainty of evidence was very low for all four outcomes (Additional file 4). Mortality was downgraded for serious risk of bias (inadequate severity adjustment in four of nine studies, unaddressed immortal time bias in three of nine) and for imprecision (confidence intervals crossing the null). ICU and hospital length of stay were further downgraded for indirectness (heterogeneous definitions, structural double-counting). Duration of mechanical ventilation (three studies) was additionally downgraded for inconsistency.

## Discussion

This systematic review, covering nine observational studies from seven countries, found that inter-hospital ICU-to-ICU transfer during the COVID-19 pandemic was not clearly associated with increased mortality. None of the adjusted effect estimates showed a statistically significant disadvantage for transferred patients; the pooled adjusted subgroup estimates (OR 1.29, 95% CI 0.84 to 1.98; HR 0.71, 95% CI 0.34 to 1.46) and a crude-effect sensitivity analysis using a common measure (OR 0.90, 95% CI 0.74 to 1.09) were all non-significant. However, their confidence intervals were wide and did not exclude clinically important harm or benefit, and the certainty of evidence was very low.

These findings are consistent with a broader body of pre-pandemic literature on inter-hospital ICU transfer in general, non-COVID populations. Golestanian et al. found that among 4,569 ICU patients, transfer patients had higher crude mortality (22% vs. 14%), but when stratified by APACHE III severity no difference in ICU or hospital mortality remained^20^. Similarly, Duke and Green reported no significant mortality difference in 73 matched transferred versus non-transferred ICU patients in Australia (24.7% vs. 17.8%; OR 1.5, 95% CI 0.68 to 3.4)^21^. Hill et al. found that after case-mix adjustment ICU mortality remained modestly elevated for transfer patients (adjusted OR 1.30, 95% CI 1.09 to 1.56), although hospital mortality did not differ significantly^22^. Rosenberg et al. reported that transfer patients had longer stays and higher crude mortality, which they attributed largely to higher illness severity and inadequate case-mix adjustment in benchmarking systems^23^. In a large propensity-matched UK cohort, Barratt et al. found that non-clinical inter-hospital ICU transfer was not associated with increased mortality^6^. Taken together, the consistent pattern across pre-pandemic and pandemic settings suggests that observed outcome differences are driven primarily by case mix rather than by the transfer process itself, although this remains an inference from observational data. The pandemic, despite its challenges of infection-control requirements, personal protective equipment constraints, and a novel disease, did not appear to fundamentally alter this relationship.

A key factor that may contribute to these reassuring findings is patient selection: those transferred had to be judged stable enough for transport. The higher proportion of transferred patients receiving invasive mechanical ventilation, despite broadly comparable severity scores, may reflect logistical factors, a closed ventilator circuit facilitates transport, and patients may be intubated in anticipation of transfer, rather than necessarily greater illness severity. A central methodological concern in transfer studies is immortal time bias, because transferred patients must survive until the moment of transfer, which creates a systematic survival advantage. Three of the reviewed studies used techniques intended to mitigate this bias (Wortel 2022^19^: left truncation; Grimaud 2026^13^: day-5 conditioning; Toss Agegard 2025^18^: landmark analyses); however, immortal time bias can rarely be fully eliminated in observational designs, and some of these approaches, by conditioning on post-transfer survival, may introduce their own selection bias. This bias operates in the opposite direction to confounding by indication, whereby sicker patients are more likely to be transferred, so the net direction of residual bias is uncertain. That the two studies with the most robust bias handling (Wortel 2022^19^: HR 0.99; Toss Agegard 2025^18^: HR 0.47) also showed no disadvantage is reassuring, though not definitive.

Although mortality did not differ clearly between groups, ICU and hospital stays were longer for transferred patients in most studies (Fig. 3). This is partly structural: in registry-based studies that capture the whole episode, transferred patients accumulate days at both the sending and receiving hospital, and preparation, transport, and the familiarisation period at the receiving centre likely contribute as well. However, length-of-stay comparisons are confounded by the reason for transfer and by differing measurement anchors across studies. In Australia, where a far higher proportion of transferred patients received ECMO (26% vs. 2%), the more than four-fold longer ICU stay^12^ more plausibly reflects the transfer of sicker patients for higher-level therapy than the transfer process itself. Longer stays for transferred patients were also reported in some pre-pandemic studies^20,23^.

Our review focused exclusively on post-transfer clinical outcomes, comparing transferred and non-transferred cohorts. A related but distinct question concerns the safety of the transport itself, which was not examined in this review. Several studies have, however, examined transport-related complications during the COVID-19 pandemic. Hilbert-Carius et al. analysed 385 COVID-19 inter-hospital and primary missions across six European air ambulance services and found that safe transport was achievable with appropriate protocols^24^. Heinrich et al. reported a very low complication rate of 1.0% among 896 helicopter transports of ventilated COVID-19 patients in Germany^25^. Slagt et al. found that although haemodynamic parameters changed during helicopter transfer of 98 ventilated COVID-19 patients, these changes were not clinically relevant^26^. Beyond COVID-19, a meta-analysis by Jeyaraju et al. of 19 studies encompassing nearly 15,000 critically ill patients estimated a pooled adverse-event rate of 11% during inter-hospital transport and concluded that adverse events were related primarily to illness severity rather than to transport itself^27^. Collectively, these findings suggest that inter-hospital transport of critically ill patients, including those with COVID-19, can be performed safely when established retrieval protocols are followed. No systematic review has yet synthesised the evidence on transport-related adverse events specifically for COVID-19 patients; such a review would complement the present work and should be a priority for future research.

Several limitations should be acknowledged. First, all included studies were observational and therefore subject to residual confounding; transferred patients represent a selected population,  sufficiently stable for transport yet requiring escalation, so confounding by indication cannot be excluded. Second, immortal time bias was addressed in only three studies, and the techniques used (left truncation, landmark analysis, day-5 conditioning) can themselves introduce selection bias by conditioning on post-transfer survival, so the net direction of residual bias is uncertain. Third, the included studies estimate different parameters in different populations, ranging from the effect of transfer at any time point (e.g., Cini 2023^12^, where transfer for ECMO may partly mark illness severity) to the effect of transfer among patients well enough to be transferred (e.g., Toss Agegard 2025^18^ and Wortel 2022^19^, whose left-truncated designs condition on survival to transfer). Fourth, studies captured different segments of the transfer pathway,bsome included only patients transferred in (e.g., Chen 2021^11^ and others only patients transferred out (e.g., Toss Agegard 2025^18^, which may introduce selection bias for a review whose exposure is inter-hospital transfer.

Fifth, transferred patients in COVID-19 ICU care likely comprised two distinct groups, a majority moved for capacity reasons (relatively stable and fit for transfer) and a minority moved to specialised centres for therapies such as ECMO (with an inherently higher risk of death), but the aggregate data are not granular enough to quantify these proportions, which limits interpretation. Sixth, treatment-limitation decisions were imbalanced in some studies, with higher rates among non-transferred patients, which could bias mortality comparisons. Seventh, at least one hazard ratio (Toss Agegard 2025^18^ rested on a Cox model whose proportional-hazards assumption did not appear to be fully met. Eighth, three French first-wave cohorts (Sanchez 2021^15^, Grimaud 2026^13^, Painvin 2021^14^ overlap in time and region, so the pooled sample cannot be interpreted as unique patients. Ninth, the diversity of effect measures (OR, HR, RR, IRR, RD) and mortality timepoints (in-hospital, ICU, 28-day, 30-day, 180-day) precluded a single pooled estimate; with few poolable studies, both the subgroup pools and the crude-effect sensitivity analysis are exploratory and underpowered, and heterogeneity statistics derived from two or three studies are unstable. Tenth, we searched three databases but did not search Embase or grey literature and did not contact study authors for raw or additional data, so a pooled analysis using individual-patient data or uniformly defined event counts was not possible. Finally, long-term functional outcomes and quality of life after transfer remain unexplored.

To our knowledge, this is the first systematic review specifically addressing inter-hospital ICU-to-ICU transfer outcomes in COVID-19 patients with a non-transferred comparator, drawing on nine studies from seven countries and diverse health systems. Its strengths include a pre-registered protocol, a reproducible search and analysis, and the deliberate avoidance of pooling incompatible effect measures.

## Conclusions

In this review of observational studies, inter-hospital ICU-to-ICU transfer of critically ill COVID-19 patients was not clearly associated with increased mortality, and transferred patients tended to have longer ICU and hospital stays. However, the certainty of this evidence is very low, and heterogeneity, residual confounding, patient selection, and immortal time bias limit causal interpretation; clinically important harm or benefit cannot be excluded. These findings are reassuring and suggest that inter-hospital transfer may be a feasible strategy for managing ICU surge capacity when performed under appropriate retrieval protocols, but they do not by themselves establish transfer as inherently safe. Future studies should standardise outcome definitions, routinely address immortal time bias, distinguish capacity-driven from clinically driven transfers, and assess long-term functional outcomes. A complementary systematic review of transport-related adverse events during inter-hospital transfer of COVID-19 patients would help complete the assessment of the entire transfer pathway.

## Supplementary Information

Below is the link to the electronic supplementary material.

Supplementary Material 1

Supplementary Material 2

Supplementary Material 3

Supplementary Material 4

Supplementary Material 5

Supplementary Material 6

Supplementary Material 7

Supplementary Material 8

Supplementary Material 9

## Data Availability

All data supporting the findings of this systematic review are available within the article and its supplementarymaterials. No primary patient-level data were generated. The extracted study-level data, full-text exclusion reasons, risk of bias assessments, and GRADE evidence profiles are provided as supplementary files. The review protocol wasprospectively registered on PROSPERO (CRD420261356915). The analysis scripts used to generate figures and tables areavailable from the corresponding author upon reasonable request.

## Abbreviations

APACHE: Acute Physiology and Chronic Health Evaluation
CI: Confidence interval
COVID-19: Coronavirus disease 2019
ECMO: Extracorporeal membrane oxygenation
GRADE: Grading of Recommendations Assessment, Development and Evaluation
HR: Hazard ratio
ICU: Intensive care unit
IRR: Incidence rate ratio
LOS: Length of stay
MV: Mechanical ventilation
NOS: Newcastle-Ottawa Scale
OR: Odds ratio
PICO: Population, Intervention, Comparison, Outcome
PRISMA: Preferred Reporting Items for Systematic Reviews and Meta-Analyses
PROSPERO: International Prospective Register of Systematic Reviews
RD: Risk difference
RR: Relative risk
SAPS: Simplified Acute Physiology Score
SARS-CoV-2: Severe acute respiratory syndrome coronavirus 2

## Declarations

### Use of large language models

During the risk-of-bias assessment, a large language model (Claude, Anthropic) was used as one of three independent assessors of the risk-of-bias items. All model-generated ratings were independently reviewed and verified by a human author (JB), who retained final responsibility for every judgment. The tool was not used for the literature search, study screening, or data extraction. No AI tool met the ICMJE authorship criteria, and the authors take full responsibility for the integrity and content of the manuscript.
