# Supplementary material for: Inter-hospital ICU-to-ICU transfer of critically ill COVID-19 patients is not associated with increased mortality: a systematic review with exploratory meta-analysis
Source: Sci Rep. 2026 Aug 8;16:24551. doi: 10.1038/s41598-026-62965-7 (PMC13452817; doi:10.1038/s41598-026-62965-7)
Supplement: Supplementary file 1 [file 41598_2026_62965_MOESM2_ESM.docx]

**Supplementary Material**

**GRADE Certainty of Evidence Assessment**

## Methods

The overall certainty of evidence for each outcome was assessed using the GRADE approach (Grading of Recommendations Assessment, Development and Evaluation), as pre-specified in the PROSPERO registration (CRD420261356915). Observational studies start at “Low” certainty and can be downgraded for five domains: risk of bias, inconsistency, indirectness, imprecision, and publication bias. Upgrading is possible for large magnitude of effect, dose-response gradient, or if all plausible residual confounders would reduce the demonstrated effect.

Evidence was rated across four levels: High, Moderate, Low, or Very Low. The assessment was informed by the Newcastle-Ottawa Scale (NOS) risk of bias results, the extracted study data, and the meta-analysis results where available.

Outcomes assessed were: mortality (primary), ICU length of stay, hospital length of stay, and duration of mechanical ventilation (all secondary), as specified in the PROSPERO protocol.

## Table G1. GRADE Summary of Findings

| **Outcome** | **Studies (n)** | **Total N** | **Summary of findings** | **Risk of bias** | **Incon- sistency** | **Indirect- ness** | **Impre- cision** | **Pub. bias** | **Certainty** |
| --- | --- | --- | --- | --- | --- | --- | --- | --- | --- |
| **Mortality (Primary)** | 9 (5 in MA) | ~35,300 | No significant difference (pooled OR 1.29 [0.84–1.98]; pooled HR 0.71 [0.34–1.46]) | **Serious (−1)** | Not serious (0) | Not serious (0) | **Serious (−1)** | Undetected (0) | **⬤○○○ Very Low** |
| **ICU Length of Stay (Secondary)** | 8 | ~35,200 | Longer in transferred (medians 10–30 vs. 5–19 days); not poolable | **Serious (−1)** | Not serious (0) | **Serious (−1)** | **Serious (−1)** | Undetected (0) | **⬤○○○ Very Low** |
| **Hospital Length of Stay (Secondary)** | 6 | ~23,200 | Longer in transferred (medians 17–37 vs. 12–20 days); not poolable | **Serious (−1)** | Not serious (0) | **Serious (−1)** | **Serious (−1)** | Undetected (0) | **⬤○○○ Very Low** |
| **MV Duration (Secondary)** | 3 | ~539 | Inconsistent: 1 study shorter, 2 studies longer for transferred; not poolable | **Serious (−1)** | **Serious (−1)** | **Serious (−1)** | **Serious (−1)** | Undetected (0) | **⬤○○○ Very Low** |

*GRADE certainty ratings: ⬤⬤⬤⬤ High; ⬤⬤⬤○ Moderate; ⬤⬤○○ Low; ⬤○○○ Very Low. Observational studies start at Low. MA = meta-analysis.*

## Detailed GRADE Evidence Profiles

**Mortality (Primary outcome)**

*Studies: 9/9 reporting | Total N: ~35,300 | Starting level: Low (observational) | 5 studies in exploratory meta-analysis*

| **Domain** | **Rating** | **Justification** |
| --- | --- | --- |
| Risk of bias | **Serious (−1)** | 4/9 studies did not adjust for disease severity (the most critical confounder due to confounding by indication). Immortal time bias not addressed in 3/9 and only partially in 3/9 studies. |
| Inconsistency | Not serious (0) | Direction of effect consistent across 6/7 adjusted estimates (no harm or benefit). OR subgroup I²=0%. HR subgroup I²=90% but unstable at k=2. |
| Indirectness | Not serious (0) | PICO alignment is direct. Mortality timepoint variation (hospital/28d/30d/180d) reflects the field, not indirectness. |
| Imprecision | **Serious (−1)** | Pooled subgroup CIs cross null (OR 0.84–1.98; HR 0.34–1.46). No overall estimate calculable. Both harm and benefit remain possible. |
| Publication bias | Undetected (0) | Not formally assessable (k<10). No obvious pattern of selective reporting. |
| **Upgrades** | None | No large effect, no dose-response, plausible confounders do not strengthen inference. |
| **FINAL CERTAINTY** | **⬤○○○ Very Low** |  |
| *Plain language* |  | *Very low certainty evidence suggests that inter-hospital ICU transfer during COVID-19 may have little or no effect on mortality, but clinically meaningful harm or benefit cannot be excluded.* |

**ICU Length of Stay (Secondary outcome)**

*Studies: 8/9 reporting | Total N: ~35,200 | Starting level: Low (observational)*

| **Domain** | **Rating** | **Justification** |
| --- | --- | --- |
| Risk of bias | **Serious (−1)** | No study reports adjusted ICU-LOS estimates. Immortal time bias inflates transferred patients’ ICU-LOS by definition (ICU time at both hospitals). Only 3/8 adequately addressed ITB. |
| Inconsistency | Not serious (0) | 7/8 studies show same direction (longer for transferred). Chen exception has wide overlapping IQRs. Magnitude variation explained by definition differences. |
| Indirectness | **Serious (−1)** | Critical heterogeneity in ICU-LOS definition (total vs. receiving-hospital only). Structural double-counting: transferred patients accumulate ICU days at two facilities. Measured outcome may not reflect the intended clinical construct. |
| Imprecision | **Serious (−1)** | No pooled estimate possible (medians, not means). Wide overlapping IQRs. Precision of overall evidence body cannot be formally evaluated. |
| Publication bias | Undetected (0) | Not assessable (k<10). ICU-LOS routinely reported as secondary outcome. |
| **Upgrades** | None | No large effect, no dose-response, plausible confounders do not strengthen inference. |
| **FINAL CERTAINTY** | **⬤○○○ Very Low** |  |
| *Plain language* |  | *Very low certainty evidence suggests transferred patients have longer ICU stays, but this likely partly reflects double-counting of ICU time across facilities rather than a true adverse effect.* |

**Hospital Length of Stay (Secondary outcome)**

*Studies: 6/9 reporting | Total N: ~23,200 | Starting level: Low (observational)*

| **Domain** | **Rating** | **Justification** |
| --- | --- | --- |
| Risk of bias | **Serious (−1)** | 2/6 reporting studies Moderate NOS. Most report only unadjusted medians. Double-counting of hospitalization days at sending and receiving hospitals is a systematic measurement bias. |
| Inconsistency | Not serious (0) | 5/6 studies show same direction (longer for transferred). Chen exception is modest (−3 days). Magnitude variation explainable. |
| Indirectness | **Serious (−1)** | Heterogeneous definitions (total vs. receiving hospital only). Only 6/9 studies report this outcome. Double-counting problem same as ICU-LOS. |
| Imprecision | **Serious (−1)** | No pooled estimate (medians vs. means). Wide IQRs. No summary effect size with precision boundaries. |
| Publication bias | Undetected (0) | Not assessable (k<10). Hospital-LOS routinely reported. |
| **Upgrades** | None | No large effect, no dose-response, plausible confounders do not strengthen inference. |
| **FINAL CERTAINTY** | **⬤○○○ Very Low** |  |
| *Plain language* |  | *Very low certainty evidence suggests transferred patients have longer hospital stays, but uncertain definitions and double-counting of hospitalization days limit interpretation.* |

**MV Duration (Secondary outcome)**

*Studies: 3/9 reporting | Total N: ~539 | Starting level: Low (observational)*

| **Domain** | **Rating** | **Justification** |
| --- | --- | --- |
| Risk of bias | **Serious (−1)** | No adjusted estimates. ITB not addressed in 2/3 studies. Unadjusted medians only. |
| Inconsistency | **Serious (−1)** | Mixed direction: Chen shorter (8 vs. 10d), Painvin longer (18 vs. 14d), Stark much longer (23 vs. 4.5d). Only 3 studies, no subgroup analysis possible. |
| Indirectness | **Serious (−1)** | Denominator problem: Painvin 100% MV in both groups (comparable), Stark all transferred but only subset of NT ventilated (conflates incidence with duration). |
| Imprecision | **Serious (−1)** | Only 3 studies, n≈539. No CIs for between-group differences. Sparse data preclude any reliable conclusion. |
| Publication bias | Undetected (0) | Not assessable (k=3). MV duration secondary outcome, less prone to selective reporting. |
| **Upgrades** | None | No large effect, no dose-response, plausible confounders do not strengthen inference. |
| **FINAL CERTAINTY** | **⬤○○○ Very Low** |  |
| *Plain language* |  | *Very low certainty evidence with inconsistent findings means we cannot determine whether inter-hospital transfer affects the duration of mechanical ventilation.* |

## Suggested Manuscript Text

*The following text is provided as a template for incorporating the GRADE assessment into the manuscript. It can be adapted as needed.*

### For Methods section:

The overall certainty of evidence for each outcome was assessed using the GRADE approach (Grading of Recommendations Assessment, Development and Evaluation). Observational studies start at “low” certainty and can be downgraded for risk of bias, inconsistency, indirectness, imprecision, and publication bias, or upgraded for large effect magnitude, dose-response gradient, or if all plausible residual confounders would reduce the demonstrated effect. The assessment was informed by the Newcastle-Ottawa Scale risk of bias results. Evidence was rated as high, moderate, low, or very low certainty for each of the four pre-specified outcomes (mortality, ICU length of stay, hospital length of stay, duration of mechanical ventilation). Results are presented in a Summary of Findings table (Supplementary Table G1).

### For Results section:

**Certainty of evidence (GRADE)**

The GRADE certainty of evidence was rated as very low for all four assessed outcomes (Supplementary Table G1). For the primary outcome of mortality, the certainty was downgraded from low (observational studies) due to serious risk of bias (inadequate adjustment for disease severity in four of nine studies and unaddressed immortal time bias in three studies) and serious imprecision (pooled subgroup confidence intervals crossing the null, with no overall pooled estimate calculable due to incompatible effect measures).

For the secondary outcomes of ICU and hospital length of stay, very low certainty was driven by the additional concern of serious indirectness: heterogeneous definitions of length of stay across studies and the structural double-counting of care days at both sending and receiving hospitals for transferred patients made it unclear to what extent observed differences reflected a true adverse effect of the transfer process versus an inherent measurement artefact.

Duration of mechanical ventilation was reported in only three studies and was additionally downgraded for serious inconsistency (mixed direction of effect) and serious indirectness (heterogeneous denominators across studies).

### For Discussion section:

The GRADE assessment rated the certainty of evidence as very low for all outcomes. This is consistent with the inherent limitations of observational transfer studies, where confounding by indication (sicker patients being selected for transfer) and immortal time bias (transferred patients must survive to the point of transfer) represent competing biases that are difficult to fully resolve. The very low certainty rating does not imply that transfer is harmful — rather, it reflects that the current evidence base cannot definitively establish the true effect of inter-hospital transfer on clinical outcomes. The consistent direction of effect across studies (no increased mortality risk) provides reassurance, but better-designed studies with standardized severity adjustment and immortal time bias handling are needed to increase the certainty of this conclusion.
