# Supplementary material for: Inter-hospital ICU-to-ICU transfer of critically ill COVID-19 patients is not associated with increased mortality: a systematic review with exploratory meta-analysis
Source: Sci Rep. 2026 Aug 8;16:24551. doi: 10.1038/s41598-026-62965-7 (PMC13452817; doi:10.1038/s41598-026-62965-7)
Supplement: Supplementary file 2 [file 41598_2026_62965_MOESM3_ESM.docx]

**Supplementary Material**

**Risk of Bias Assessment — Newcastle-Ottawa Scale (NOS)**

## Methods

Risk of bias was assessed using the Newcastle-Ottawa Scale (NOS) for cohort studies, as pre-specified in the PROSPERO registration (CRD420261356915). The NOS evaluates three domains: Selection (4 items, max 4 stars), Comparability (1 item, max 2 stars), and Outcome (3 items, max 3 stars), for a maximum of 9 stars.

Studies were categorized as: Low risk of bias (7–9 stars), Moderate risk of bias (4–6 stars), or High risk of bias (0–3 stars).

For the Comparability domain, the two most important confounders were pre-specified as: (a) age — the strongest predictor of COVID-19 mortality, and (b) disease severity (APACHE, SAPS, SOFA, or clinical proxy such as mechanical ventilation status) — critical due to confounding by indication, whereby sicker patients are more likely to be transferred.

Additionally, the presence or absence of methods to address immortal time bias (ITB) was specifically assessed for each study, given its particular relevance to transfer studies: transferred patients must, by definition, survive until the moment of transfer.

Assessment was performed independently by two reviewers (Reviewer 1: AI-assisted using Claude; Reviewer 2: human), with discrepancies resolved through discussion, as pre-specified in the PROSPERO protocol.

## Table S1. Risk of Bias Summary — Newcastle-Ottawa Scale

| **Study** | **S1** | **S2** | **S3** | **S4** | **C1a** | **C1b** | **O1** | **O2** | **O3** | **Total** | **Category** |
| --- | --- | --- | --- | --- | --- | --- | --- | --- | --- | --- | --- |
| Wortel 2022 | **★** | **★** | **★** | **★** | **★** | **★** | **★** | **★** | **★** | **9/9** | **Low** |
| Grimaud 2026 | **★** | **★** | **★** | **★** | **★** | **★** | **★** | **★** | **★** | **9/9** | **Low** |
| Cini 2023 | **★** | **★** | **★** | **★** | **★** | **★** | **★** | **★** | **★** | **9/9** | **Low** |
| Sanchez 2021 | **★** | **★** | **★** | **★** | **★** | **–** | **★** | **★** | **★** | **8/9** | **Low** |
| Toss Agegard 2025 | **–** | **★** | **★** | **★** | **★** | **★** | **★** | **★** | **★** | **8/9** | **Low** |
| Stark 2023 | **–** | **★** | **★** | **★** | **★** | **★** | **★** | **★** | **★** | **8/9** | **Low** |
| Chen 2021 | **–** | **★** | **★** | **★** | **★** | **–** | **★** | **★** | **★** | **7/9** | **Low** |
| Suski 2024 | **–** | **★** | **★** | **★** | **–** | **–** | **★** | **★** | **★** | **6/9** | **Moderate** |
| Painvin 2021 | **–** | **–** | **★** | **★** | **–** | **–** | **★** | **★** | **★** | **5/9** | **Moderate** |

*S1 = Representativeness of exposed cohort; S2 = Selection of non-exposed; S3 = Ascertainment of exposure; S4 = Outcome not present at start; C1a = Controls for age; C1b = Controls for disease severity; O1 = Assessment of outcome; O2 = Follow-up duration; O3 = Adequacy of follow-up.*

*★ = star awarded (low risk); – = no star (high risk for that item). Low risk: 7–9 stars; Moderate risk: 4–6 stars; High risk: 0–3 stars.*

## Table S2. Immortal Time Bias Assessment

| **Study** | **n (T / NT)** | **Effect Measure** | **ITB Assessment** | **Method / Details** |
| --- | --- | --- | --- | --- |
| Wortel 2022 | 2,814 / 7,395 | HR 0.99 (0.91–1.08) | **Addressed** | Left truncation in Cox model |
| Grimaud 2026 | 285 / 667 | IRR 0.14 (0.10–0.19) | **Addressed** | Controls alive at day 5 post-transfer; sensitivity at day 3 and 7 |
| Cini 2023 | 328 / 4,879 | RD −5.0pp (−10 to −0.03) | **Partially** | Propensity score sensitivity analysis |
| Sanchez 2021 | 2,228 / 15,303 | RR 0.38–0.98 (range) | **Partially** | Sensitivity KM with start-time at transfer or Day 5 |
| Toss Agegard 2025 | 133 / 532 | HR 0.47 (0.30–0.76) | **Addressed** | Landmark sensitivity (>6, >8, >10, >12 days ICU LOS) |
| Stark 2023 | 30 / 78 | OR 0.88 (inv.) | **Not addressed** | No method applied; median 6 days to transfer |
| Chen 2021 | 117 / 181 | OR 1.45 (0.86–2.43) | **Partially** | D0 = first ventilation day or ICU admission (partial alignment) |
| Suski 2024 | 115 / 100 | p = 0.663 (unadj.) | **Not addressed** | No method applied; not discussed |
| Painvin 2021 | 68 / 65 | OR 1.20 (0.35–4.09) | **Not addressed** | No method applied; median 7.5 days to transfer |

*Addressed = explicit method applied (landmark, left-truncation, time-dependent exposure, survival-time conditioning). Partially = sensitivity analysis or partial consideration. Not addressed = no method applied or discussed.*

## Table S3. Study Characteristics and Risk of Bias Assessment

| **Study** | **Country** | **Design** | **n (T / NT)** | **Mortality Endpoint** | **Effect (95% CI)** | **NOS Score** | **Category** | **ITB** |
| --- | --- | --- | --- | --- | --- | --- | --- | --- |
| Wortel 2022 | Netherlands | Nationwide registry (73 ICUs) | 2,814 / 7,395 | 180-day | HR 0.99 (0.91–1.08) | **9/9** | **Low** | **Addressed** |
| Grimaud 2026 | France | Multi-center matched (111 hosp.) | 285 / 667 | 28-day ICU | IRR 0.14 (0.10–0.19) | **9/9** | **Low** | **Addressed** |
| Cini 2023 | Australia | National registry (63 ICUs) | 328 / 4,879 | Hospital | RD −5.0pp (−10 to −0.03) | **9/9** | **Low** | **Partially** |
| Sanchez 2021 | France | Nationwide registry (SI-VIC) | 2,228 / 15,303 | Hospital | RR 0.38–0.98 (range) | **8/9** | **Low** | **Partially** |
| Toss Agegard 2025 | Sweden | Single-center | 133 / 532 | 30-day | HR 0.47 (0.30–0.76) | **8/9** | **Low** | **Addressed** |
| Stark 2023 | UK | Single-center | 30 / 78 | Hospital | OR 0.88 (inv.) | **8/9** | **Low** | **Not addressed** |
| Chen 2021 | USA | Single-center | 117 / 181 | Hospital | OR 1.45 (0.86–2.43) | **7/9** | **Low** | **Partially** |
| Suski 2024 | Germany | Regional (4 hospitals) | 115 / 100 | Hospital | p = 0.663 (unadj.) | **6/9** | **Moderate** | **Not addressed** |
| Painvin 2021 | France | Multi-center (3 hosp.) | 68 / 65 | Hospital | OR 1.20 (0.35–4.09) | **5/9** | **Moderate** | **Not addressed** |

## Table S4. Detailed NOS Justifications per Study

**Wortel 2022 — 9/9 stars (Low risk)**

| **Domain** | **Item** | **Rating** | **Justification** |
| --- | --- | --- | --- |
| Selection | **S1** | **★** | NICE national registry covering all 73 Dutch ICUs — population-based multi-center sample. |
| Selection | **S2** | **★** | Non-transferred from same NICE registry, same nationwide ICU population, same period. |
| Selection | **S3** | **★** | NICE registry COVID-19 module tracking consecutive ICU admissions/discharges. |
| Selection | **S4** | **★** | All alive at ICU admission; left truncation ensures entry alive at transfer day. |
| Comparability | **C1a** | **★** | Age modeled as spline in Cox regression, identified via DAG analysis. |
| Comparability | **C1b** | **★** | APACHE IV mortality probability (spline), MV first 24h, vasoactive medication. |
| Outcome | **O1** | **★** | NICE registry + Vektis national insurance database for post-discharge deaths. |
| Outcome | **O2** | **★** | 180-day follow-up after ICU admission. |
| Outcome | **O3** | **★** | Vektis covers 97% of Dutch population; 4/10,213 excluded for missing data. |

*Immortal time bias: Addressed — Left truncation in Cox model*

**Grimaud 2026 — 9/9 stars (Low risk)**

| **Domain** | **Item** | **Rating** | **Justification** |
| --- | --- | --- | --- |
| Selection | **S1** | **★** | Multi-center across 111 hospitals; near-population-based sample of national mass evacuation. |
| Selection | **S2** | **★** | Controls from same origin ICUs, admitted within ±2 days of index patient. |
| Selection | **S3** | **★** | ICU health records and eCRF at both origin and destination hospitals. |
| Selection | **S4** | **★** | All alive at day 5 post-transfer — ensured by study design. |
| Comparability | **C1a** | **★** | Exact matching (same ICU, admission ±2 days), propensity score, adjusted Poisson. |
| Comparability | **C1b** | **★** | SAPS II in PS model + Knaus scale, CFS, ventilation mode, lung lesion extent, lactate. |
| Outcome | **O1** | **★** | ICU health records at origin and destination; vital status recorded in eCRF. |
| Outcome | **O2** | **★** | 28-day follow-up after transfer date. |
| Outcome | **O3** | **★** | 285/328 (87%) transferred matched; complete vital status for all 952 included. |

*Immortal time bias: Addressed — Controls alive at day 5 post-transfer; sensitivity at day 3 and 7*

**Cini 2023 — 9/9 stars (Low risk)**

| **Domain** | **Item** | **Rating** | **Justification** |
| --- | --- | --- | --- |
| Selection | **S1** | **★** | SPRINT-SARI Australia national registry, 63 ICUs — population-based consecutive sample. |
| Selection | **S2** | **★** | Non-transferred from same registry and same 63 ICUs, same period. |
| Selection | **S3** | **★** | Prospectively recorded in SPRINT-SARI database with separate transfer log. |
| Selection | **S4** | **★** | All alive at ICU admission. |
| Comparability | **C1a** | **★** | Age adjusted in multivariable regression and propensity score model. |
| Comparability | **C1b** | **★** | APACHE II adjusted, plus MV status and co-existing disorders. |
| Outcome | **O1** | **★** | Hospital records and registry database, collected by dedicated research staff. |
| Outcome | **O2** | **★** | Follow-up to hospital discharge; KM curves to day 90. |
| Outcome | **O3** | **★** | Outcome data for 4,796/5,207 (92%); missing addressed via multiple imputation. |

*Immortal time bias: Partially — Propensity score sensitivity analysis*

**Sanchez 2021 — 8/9 stars (Low risk)**

| **Domain** | **Item** | **Rating** | **Justification** |
| --- | --- | --- | --- |
| Selection | **S1** | **★** | SI-VIC national registry capturing all COVID-19 hospitalizations in France. |
| Selection | **S2** | **★** | Non-transferred from same SI-VIC registry, same period and country. |
| Selection | **S3** | **★** | SI-VIC registry with unique anonymous patient identifiers tracking all transfers. |
| Selection | **S4** | **★** | All alive at ICU admission. |
| Comparability | **C1a** | **★** | Modified Poisson regression adjusted for age (seven categorical strata). |
| Comparability | **C1b** | **–** | No severity score available (APACHE/SAPS/SOFA). Authors acknowledged this as main limitation. |
| Outcome | **O1** | **★** | SI-VIC registry hospital records with mandatory standardized reporting. |
| Outcome | **O2** | **★** | Follow-up until 14 July 2020; only 0.9% still in ICU at end. |
| Outcome | **O3** | **★** | 817/18,348 (4.5%) excluded for incomplete data; <2% lost to follow-up. |

*Immortal time bias: Partially — Sensitivity KM with start-time at transfer or Day 5*

**Toss Agegard 2025 — 8/9 stars (Low risk)**

| **Domain** | **Item** | **Rating** | **Justification** |
| --- | --- | --- | --- |
| Selection | **S1** | **–** | Single-center (Södersjukhuset, Stockholm) — not population-based or multi-center. |
| Selection | **S2** | **★** | Non-transferred from same two ICUs at same hospital, same period. |
| Selection | **S3** | **★** | Patient data management system and medical records; cross-validated by two researchers. |
| Selection | **S4** | **★** | All alive at ICU admission. |
| Comparability | **C1a** | **★** | Age adjusted as continuous variable in multivariable Cox regression. |
| Comparability | **C1b** | **★** | SAPS III (<50, 50–59, ≥60) + Charlson Comorbidity Index, invasive MV, treatment limitations. |
| Outcome | **O1** | **★** | Hospital records; Sweden has comprehensive death registration. |
| Outcome | **O2** | **★** | 30-day and 90-day follow-up from ICU admission. |
| Outcome | **O3** | **★** | Complete follow-up for all 665 patients at both timepoints. |

*Immortal time bias: Addressed — Landmark sensitivity (>6, >8, >10, >12 days ICU LOS)*

**Stark 2023 — 8/9 stars (Low risk)**

| **Domain** | **Item** | **Rating** | **Justification** |
| --- | --- | --- | --- |
| Selection | **S1** | **–** | Single-center (University Hospital Monklands, Scotland) — convenience sample. |
| Selection | **S2** | **★** | Non-transferred from same ICU at same hospital, same period. |
| Selection | **S3** | **★** | SICSAG/WardWatcher national ICU audit system and transfer documentation. |
| Selection | **S4** | **★** | All alive at ICU admission. |
| Comparability | **C1a** | **★** | Age retained in backward stepwise logistic regression (OR 0.95, 0.90–0.99). |
| Comparability | **C1b** | **★** | APACHE II retained in final model (OR 0.92, 0.83–1.0). |
| Outcome | **O1** | **★** | Hospital records and SICSAG/WardWatcher database. |
| Outcome | **O2** | **★** | Follow-up to ultimate hospital discharge. |
| Outcome | **O3** | **★** | Complete data for 101/108 (93.5%) in regression; all 108 had outcome data. |

*Immortal time bias: Not addressed — No method applied; median 6 days to transfer*

**Chen 2021 — 7/9 stars (Low risk)**

| **Domain** | **Item** | **Rating** | **Justification** |
| --- | --- | --- | --- |
| Selection | **S1** | **–** | Single-center (Rush University Medical Center) — not population-based. |
| Selection | **S2** | **★** | Non-transferred (ED admits) from same institution, same period. |
| Selection | **S3** | **★** | EMR, validated by dual-physician chart review. |
| Selection | **S4** | **★** | All alive at ICU admission. |
| Comparability | **C1a** | **★** | Age in multivariable logistic regression and IPTW propensity score model. |
| Comparability | **C1b** | **–** | No validated severity score (APACHE/SAPS/SOFA). Comorbidities controlled but not acute severity. |
| Outcome | **O1** | **★** | Hospital records (EMR), including discharge to hospice. |
| Outcome | **O2** | **★** | Follow-up to hospital discharge. |
| Outcome | **O3** | **★** | Complete follow-up for all 298 patients; <2% excluded for missing demographics only. |

*Immortal time bias: Partially — D0 = first ventilation day or ICU admission (partial alignment)*

**Suski 2024 — 6/9 stars (Moderate risk)**

| **Domain** | **Item** | **Rating** | **Justification** |
| --- | --- | --- | --- |
| Selection | **S1** | **–** | Regional 4-hospital network (RoMed, Bavaria) — geographically limited convenience sample. |
| Selection | **S2** | **★** | Non-transferred from same 4-hospital network, same period. |
| Selection | **S3** | **★** | Hospital medical files with detailed transfer documentation. |
| Selection | **S4** | **★** | All alive at hospital admission. |
| Comparability | **C1a** | **–** | Mortality comparison was univariate only (p=0.663). Age adjusted only for LOS, not mortality. |
| Comparability | **C1b** | **–** | No adjusted mortality analysis. Simplified APACHE-II computed but not used for mortality model. |
| Outcome | **O1** | **★** | Hospital records; available for 759/760 patients. |
| Outcome | **O2** | **★** | Follow-up to hospital discharge. |
| Outcome | **O3** | **★** | Mortality data for 759/760 (99.9%). |

*Immortal time bias: Not addressed — No method applied; not discussed*

**Painvin 2021 — 5/9 stars (Moderate risk)**

| **Domain** | **Item** | **Rating** | **Justification** |
| --- | --- | --- | --- |
| Selection | **S1** | **–** | Three receiving hospitals; transferred cohort explicitly described as “selected” by authors. |
| Selection | **S2** | **–** | Non-transferred locally admitted in West France, not from same source population (Paris/East). |
| Selection | **S3** | **★** | Hospital medical records across three university hospitals. |
| Selection | **S4** | **★** | All 133 patients alive at ICU admission; no deaths during evacuation. |
| Comparability | **C1a** | **–** | Model adjusted for AKI, proning, MV duration — but NOT for age. |
| Comparability | **C1b** | **–** | No severity score. Covariates (AKI, proning, MV duration) are mediators, not confounders. |
| Outcome | **O1** | **★** | ICU and hospital mortality from hospital records. |
| Outcome | **O2** | **★** | Follow-up through hospital discharge. |
| Outcome | **O3** | **★** | All 133 patients had complete outcome data. |

*Immortal time bias: Not addressed — No method applied; median 7.5 days to transfer*

## Summary

Of the nine included studies, seven (78%) were rated as low risk of bias (7–9 stars) and two (22%) as moderate risk of bias (5–6 stars). No study was rated as high risk of bias.

The Outcome domain was the strongest: all nine studies (100%) received full stars for outcome assessment, follow-up duration, and follow-up adequacy. The Comparability domain was the weakest: four studies (44%) did not adjust for disease severity (Chen, Sanchez, Suski, Painvin), and two of these also did not adjust for age (Suski, Painvin). In the Selection domain, five studies (56%) lost the representativeness star due to single-center or regional designs.

Regarding immortal time bias — a critical concern in transfer studies, as transferred patients must survive until the moment of transfer — three studies addressed it explicitly (Wortel: left truncation; Grimaud: day-5 conditioning; Toss Agegard: landmark sensitivity analyses), three partially addressed it (Chen, Cini, Sanchez), and three did not address it (Painvin, Stark, Suski).
