# Supplementary material for: Inter-hospital ICU-to-ICU transfer of critically ill COVID-19 patients is not associated with increased mortality: a systematic review with exploratory meta-analysis
Source: Sci Rep. 2026 Aug 8;16:24551. doi: 10.1038/s41598-026-62965-7 (PMC13452817; doi:10.1038/s41598-026-62965-7)
Supplement: Supplementary file 4 [file 41598_2026_62965_MOESM5_ESM.docx]

**Additional file 1. Full search strategies**

Three electronic databases — PubMed, Web of Science, and Scopus — were searched on 15 February 2026. The search combined three concept blocks (COVID-19; inter-hospital transfer/transport; intensive care/hospital) with the Boolean operator AND; terms within each block were combined with OR. Database-specific syntax was adapted for each database (MeSH terms and [tiab] title/abstract tags for PubMed; TS= topic search for Web of Science; TITLE-ABS-KEY for Scopus). No language restriction was applied at the search stage; eligibility was subsequently restricted to English- or German-language reports. Reference lists of all included studies were hand-searched for additional records.

**Records identified per database**

| **Source** | **Records** |
| --- | --- |
| PubMed | 6,922 |
| Web of Science | 6,732 |
| Scopus | 7,382 |
| Total records identified | 21,036 |
| Duplicate records removed | 14,214 |
| Unique records screened | 6,822 |

**PubMed**

```
#1  "COVID-19"[Mesh] OR "SARS-CoV-2"[Mesh] OR COVID*[tiab] OR
    "SARS-CoV-2"[tiab] OR "corona virus"[tiab] OR coronavirus[tiab]

#2  "Patient Transfer"[Mesh] OR "Transportation of Patients"[Mesh] OR
    transfer*[tiab] OR transport*[tiab] OR relocation*[tiab] OR
    referral*[tiab] OR evacuation*[tiab] OR aeromedical[tiab] OR
    interfacility[tiab] OR "inter-facility"[tiab] OR interhospital[tiab] OR
    "inter-hospital"[tiab] OR retrieval[tiab]

#3  "Intensive Care Units"[Mesh] OR "Critical Care"[Mesh] OR
    "Hospitals"[Mesh] OR hospital*[tiab] OR ICU*[tiab] OR
    "intensive care"[tiab] OR "critical care"[tiab] OR
    "intensive care unit*"[tiab]

#4  #1 AND #2 AND #3
```

**Web of Science**

```
#1  TS=(COVID* OR "SARS-CoV-2" OR coronavirus OR "corona virus")

#2  TS=(transfer* OR transport* OR relocation* OR referral* OR
       evacuation* OR aeromedical OR interfacility OR "inter-facility" OR
       interhospital OR "inter-hospital" OR retrieval)

#3  TS=(hospital* OR ICU* OR "intensive care" OR "critical care" OR
       "intensive care unit*")

#4  #1 AND #2 AND #3
```

**Scopus**

```
TITLE-ABS-KEY(
    (COVID* OR "SARS-CoV-2" OR coronavirus OR "corona virus")
    AND
    (transfer* OR transport* OR relocation* OR referral* OR evacuation* OR
     aeromedical OR interfacility OR "inter-facility" OR interhospital OR
     "inter-hospital" OR retrieval)
    AND
    (hospital* OR ICU* OR "intensive care" OR "critical care" OR
     "intensive care unit*")
)
AND PUBYEAR > 2019
```

*Duplicate records were removed prior to screening (14,214 duplicates), leaving 6,822 unique records for title/abstract screening (21,036 − 14,214 = 6,822).*
