# Supplementary material for: Inter-hospital ICU-to-ICU transfer of critically ill COVID-19 patients is not associated with increased mortality: a systematic review with exploratory meta-analysis
Source: Sci Rep. 2026 Aug 8;16:24551. doi: 10.1038/s41598-026-62965-7 (PMC13452817; doi:10.1038/s41598-026-62965-7)
Supplement: Supplementary file 5 [file 41598_2026_62965_MOESM6_ESM.docx]

**Additional file 7. ROBINS-I risk-of-bias assessment (primary outcome: mortality)**

The Newcastle-Ottawa Scale was retained as the pre-registered tool; this ROBINS-I assessment was added during revision because non-randomised studies of patient transfer involve time-varying exposure, confounding by indication, and selective transfer of transport-stable patients that the Newcastle-Ottawa Scale captures incompletely. Seven domains were judged (Low / Moderate / Serious / Critical / No information), with an overall judgment. Assessments were drafted with AI assistance (Claude) and verified by the authors.

| **Study** | **D1** | **D2** | **D3** | **D4** | **D5** | **D6** | **D7** | **Overall** |
| --- | --- | --- | --- | --- | --- | --- | --- | --- |
| Chen 2021 | **Serious** | **Serious** | **Low** | **Moderate** | **Moderate** | **Low** | **Low** | **Serious** |
| Cini 2023 | **Serious** | **Moderate** | **Low** | **Low** | **Moderate** | **Low** | **Low** | **Serious** |
| Grimaud 2026 | **Serious** | **Moderate** | **Low** | **Low** | **Moderate** | **Low** | **Low** | **Serious** |
| Painvin 2021 | **Serious** | **Serious** | **Low** | **Low** | **Moderate** | **Low** | **Moderate** | **Serious** |
| Sanchez 2021 | **Serious** | **Serious** | **Low** | **Low** | **Moderate** | **Low** | **Moderate** | **Serious** |
| Stark 2023 | **Serious** | **Serious** | **Low** | **Low** | **Moderate** | **Low** | **Moderate** | **Serious** |
| Suski 2024 | **Serious** | **Serious** | **Low** | **Moderate** | **Moderate** | **Low** | **Moderate** | **Serious** |
| Toss Agegard 2025 | **Serious** | **Serious** | **Low** | **Low** | **Moderate** | **Low** | **Low** | **Serious** |
| Wortel 2022 | **Moderate** | **Low** | **Low** | **Low** | **Low** | **Low** | **Low** | **Moderate** |

**Domains:** *D1 Confounding; D2 Selection of participants into the study; D3 Classification of interventions; D4 Deviations from intended interventions; D5 Missing data; D6 Measurement of outcomes; D7 Selection of the reported result.*

**Domain-level rationales**

**Chen 2021 — Overall: Serious**

- **D1 Confounding: Serious.** Classic confounding by indication — transfers were selected for surge capacity/ECMO and were more severely ill (higher d-dimer, troponin), yet validated severity scores (APACHE/SAPS) could not be used due to missing data, so adjustment relied on demographics, comorbidities, admission month, IPTW and lab surrogates, leaving substantial residual severity confounding.
- **D2 Selection of participants: Serious.** Immortal-time/survivor selection is unaddressed at the exposure boundary: transferred patients had to survive at the referring hospital long enough to be accepted and transported (transport-stable selection), and the cohort required survival-to-discharge-eligibility; deaths before/during transfer are structurally excluded and follow-up anchoring at RUMC arrival does not recover them.
- **D3 Classification of intervention: Low.** Transfer status (interhospital transfer vs ED admit) was objectively classified from EMR admission-source data at the start of follow-up, with no risk of misclassification influenced by the outcome.
- **D4 Deviations from intervention: Moderate.** Transfer itself occurred as intended with no crossover, but transfers received systematically more aggressive co-interventions (ECMO 12% vs 3%, less hospice) that differ from the exposure of interest and could bias the effect of 'transfer' per se.
- **D5 Missing data: Moderate.** Mortality outcome was essentially complete and only 5/298 (missing race/ethnicity) were dropped from regressions with a documented complete-vs-missing comparison, but severity data (physiology scores, pre-transfer ICU/transport/intubation timing) were substantially missing, limiting confounder control.
- **D6 Measurement of outcome: Low.** In-hospital mortality is an objective, hard endpoint ascertained identically from the EMR for both groups, with no plausible differential or observer-influenced measurement error.
- **D7 Selection of reported result: Low.** The primary outcome was pre-specified, STROBE-adherent reporting was followed, and results are presented consistently across unadjusted, adjusted, IPTW-weighted and Cox analyses, with no evidence of selective outcome or analysis reporting (though no public protocol is cited).

**Cini 2023 — Overall: Serious**

- **D1 Confounding: Serious.** Confounding by indication is strong and only partially controlled: transfer was driven by clinical need/higher-level care (ECMO 26% vs 1.7%, invasive ventilation 71.2% vs 38.1%), and although models adjusted for age, BMI, APACHE II, comorbidity count, hospital-to-ICU time, mechanical ventilation, admission volume and used propensity scores, the authors explicitly state data limitations precluded adjusting for illness severity at the time of transfer, clinical indication for transfer, and bed occupancy.
- **D2 Selection of participants: Moderate.** Transfer is a time-varying exposure treated as a fixed baseline group (transferred patients must survive long enough to be transferred, and pre-transfer deaths at referring hospitals may be misclassified), producing residual immortal-time/selection bias; this is partially mitigated because follow-up and LOS are anchored at the index (first) hospital admission rather than at arrival at the receiving centre, and the authors themselves acknowledge transferred patients were subject to selection bias.
- **D3 Classification of intervention: Low.** Inter-hospital transfer status was prospectively recorded for each patient and independently cross-checked via a dedicated coordinating-centre transfer log, so exposure classification is well defined and determined independently of the outcome.
- **D4 Deviations from intervention: Low.** This is an observational registry of usual care with no protocol-defined intervention to deviate from; the more intensive co-interventions received by transferred patients (ECMO, RRT, tracheostomy) are intrinsic to the higher-level-care transfer pathway being evaluated rather than external deviations.
- **D5 Missing data: Moderate.** Missingness is non-trivial and variable-specific (e.g., BMI, APACHE II, several interventions, and the outcome itself: hospital-death data available for 309/328 transferred and 4487/4879 non-transferred), but the authors addressed it with multiple imputation and complete-case sensitivity analyses that yielded broadly consistent, if attenuated, results.
- **D6 Measurement of outcome: Low.** In-hospital mortality is an objective, hard endpoint ascertained identically from medical records/registry for both groups and is not susceptible to assessor knowledge of transfer status.
- **D7 Selection of reported result: Low.** The pre-specified primary outcome (in-hospital mortality) and secondary outcomes are reported, and a full matrix of analyses (unadjusted, primary adjusted, model I, model II, propensity score, each with and without multiple imputation and with/without withdrawal-of-support exclusion) is transparently tabulated in Box 6; STROBE-adherent reporting, though no separately published pre-registered protocol is cited.

**Grimaud 2026 — Overall: Serious**

- **D1 Confounding: Serious.** Despite matching (same ICU/date), restriction to transfer-eligible controls, adjustment for age/comorbidities/SAPS II, and PS weighting, the authors concede clinical status at transfer time was never measured in controls and that clinicians selected the most stable, better-prognosis patients — unmeasured confounding by illness acuity plausibly drives the implausibly large IRR of 0.14.
- **D2 Selection of participants: Moderate.** Immortal time / time-varying exposure is deliberately handled by requiring both groups to be alive at day 5 post-transfer and starting follow-up at that anchor, but transferred patients were restricted to the 328/502 with complete origin+destination data and controls are a heavily selected matched subset, so residual selection remains.
- **D3 Classification of intervention: Low.** Transfer status is an unambiguous, record-documented event classified independently of outcome, with negligible misclassification risk.
- **D4 Deviations from intervention: Low.** This is a natural-experiment capacity-driven evacuation with no protocolised co-intervention to deviate from; both groups received standard ICU care and no outcome-related deviations are described.
- **D5 Missing data: Moderate.** Missingness was <10% for most variables (SAPS II 10.4%; plateau pressure 45.6%, tidal volume 20.3%) and multiple imputation plus complete-case sensitivity analyses gave consistent results, but ~35% of enrolled transferred patients were dropped for incomplete data (shown comparable on measured profiles).
- **D6 Measurement of outcome: Low.** Vital status (ICU death) is an objective outcome ascertained identically from medical records for both groups, unlikely to be influenced by exposure knowledge.
- **D7 Selection of reported result: Low.** The 28-day ICU case-fatality primary outcome and analytic approach were pre-specified in a published protocol (BMJ Open 2021), and crude, adjusted, PS-weighted, subgroup, and multiple sensitivity analyses are all reported consistently.

**Painvin 2021 — Overall: Serious**

- **D1 Confounding: Serious.** Transfer exposure was driven by non-random selection with likely confounding by indication/selective transfer of transport-stable patients ("transferred patients have probably been selected", "most transferred patients were clinically stable before departure"), and the adjustment set (AKI, proning sessions, MV duration) consists largely of post-baseline mediators rather than a comprehensive set of baseline confounders, although initial-admission SAPS II and SOFA were similar between groups.
- **D2 Selection of participants: Serious.** Transferred patients had to survive their sending-ICU stay (median 7.5 days) to be eligible for transfer and inclusion at the receiving centres, introducing immortal-time/survival bias and misaligned time-zero between groups (local ICU admission for non-transferred vs post-transfer arrival for transferred), while patients who died before transfer are unobserved.
- **D3 Classification of intervention: Low.** The transfer exposure is clearly and objectively defined and ascertained from medical records based on whether an inter-hospital transfer occurred, and cannot have been influenced by knowledge of the outcome.
- **D4 Deviations from intervention: Low.** The intervention is a one-time transfer event with no meaningful post-exposure co-intervention imbalance described that would systematically bias the effect estimate beyond the exposure itself.
- **D5 Missing data: Moderate.** The authors acknowledge retrospective data collection with risk of information loss when patients' medical files were transferred, and explicitly note missing pre-transportation data (e.g., neuromuscular-blocker duration excluded transferred patients' pre-transfer data), although the mortality outcome itself appears complete for the 133 patients.
- **D6 Measurement of outcome: Low.** ICU and hospital mortality are objective, hard endpoints obtained identically from records for both groups and are not susceptible to assessor influence by exposure status.
- **D7 Selection of reported result: Moderate.** The primary objective (ICU mortality) was clearly prespecified and all primary/secondary outcomes are reported in Table 1, but there is no registered protocol/analysis plan, and the confounder set for the single adjusted model is not justified a priori, leaving some risk of selective analysis reporting.

**Sanchez 2021 — Overall: Serious**

- **D1 Confounding: Serious.** The authors explicitly state the transferred and non-transferred groups 'were not comparable' and that severity scores and numerous clinical variables were unavailable, so the adjusted model (age, sex, admission period, high-incidence region, hospital type, pre-ICU location only) cannot control confounding by indication, and preferential selection of younger, clinically stable patients for transfer biases the comparison in favour of transfer.
- **D2 Selection of participants: Serious.** Transfer is a time-varying exposure that can only occur in patients who survive to be transferred (median 5 days in ICU before transfer), producing immortal time bias; this is only partially mitigated by a Day-5 landmark sensitivity analysis (Table 3/Fig 3) while the primary Kaplan–Meier and regression analyses count survival from acute-care admission.
- **D3 Classification of intervention: Low.** Transfer status (transferred to another ICU vs remaining in the initial unit) was objectively and prospectively recorded in the national registry, independent of the outcome and not subject to recall.
- **D4 Deviations from intervention: Low.** The intended intervention is the transfer strategy itself (including care in a less-saturated receiving ICU with better staffing ratios), and the exposure reflects what actually occurred; no differential protocol deviation from the transfer strategy is described, although care environments differ by design as part of the intervention.
- **D5 Missing data: Moderate.** Complete-case analysis excluding ~1,600 ICU admissions for incomplete data (817), loss to follow-up (328 in ICU, 265 post-ICU), missing age/sex (216), and out-of-window admission (8); overall ~95.6% completeness is reasonable but potential differential missingness (e.g., outcomes not updated by hospital staff) is not examined.
- **D6 Measurement of outcome: Low.** In-hospital mortality is an objective, hard outcome uniformly ascertained from the national registry for both groups and cannot be influenced by knowledge of transfer status.
- **D7 Selection of reported result: Moderate.** No pre-registered protocol/statistical analysis plan for this specific analysis is cited, and the transfer effect is presented only as distance-stratified estimates (no single transfer-vs-no-transfer effect), which emphasizes the strong long-distance association; however, all distance categories plus crude, adjusted, and Day-5 sensitivity models are reported.

**Stark 2023 — Overall: Serious**

- **D1 Confounding: Serious.** Confounding by indication is strong and only partially controlled — patients were hand-picked for transfer on the basis of clinical stability (potentially less severe/further into disease) yet were significantly older, and adjustment was limited to age, ethnicity and APACHE II via a small stepwise model while disease severity/physiology and staffing at transfer were explicitly unmeasured.
- **D2 Selection of participants: Serious.** Immortal time / time-varying-exposure bias: exposure was classified at transfer (median day 6, IQR 3-10, up to day 26 after ICU admission), so transferred patients had to survive long enough to be transferred while patients dying earlier could only fall in the non-transfer group, biasing the comparison in favour of transfer, and follow-up start (ICU admission) did not coincide with exposure onset.
- **D3 Classification of intervention: Low.** The capacity-transfer exposure is objectively and prospectively documented (standardised transfer paperwork collated by a transfer lead) based on unit bed/staff shortage, recorded at the time of transfer and independent of the survival outcome.
- **D4 Deviations from intervention: Low.** Transfer was a standardised one-off intervention (SOP, checklists, competent escort) and any peri-transfer co-interventions (e.g., deeper sedation/neuromuscular blockade) are intrinsic to the intervention rather than protocol deviations arising from the study context.
- **D5 Missing data: Moderate.** The primary survival outcome appears complete for all 108 patients, but the adjusted analysis was complete-case (72/78 non-transfers and 29/30 transfers with complete covariate data, ~6.5% missing) with no imputation or sensitivity analysis described.
- **D6 Measurement of outcome: Low.** Survival to hospital discharge is an objective, hard endpoint ascertained identically from the audit database with all patients followed to completion of their hospital stay, unlikely to be influenced by knowledge of transfer status (minor residual concern about tracking final vital status of patients discharged onward to receiving hospitals).
- **D7 Selection of reported result: Moderate.** The primary outcome was clearly pre-stated, but the adjusted estimate came from a data-driven backward stepwise variable-selection procedure in a small sample with no registered protocol, giving some scope for analysis-dependent reporting.

**Suski 2024 — Overall: Serious**

- **D1 Confounding: Serious.** Transfer was driven by capacity and medical (confounding-by-indication) reasons and transferred ICU patients differed clinically (lower Horovitz score, more invasive ventilation/ECMO); crucially, the transferred-vs-non-transferred mortality comparison is crude/unadjusted (multivariable regression was applied only to length of stay, not mortality), and the simplified APACHE-II severity score was based on incomplete data.
- **D2 Selection of participants: Serious.** Exposure was defined as 'ever transferred' (classified by the first transfer) with follow-up beginning at admission, so transferred patients had to survive at the index/sending unit long enough to be transferred (immortal-time / time-varying exposure treated as baseline), and clinicians preferentially transferred hemodynamically stable ICU patients expected to have a beneficial outcome.
- **D3 Classification of intervention: Low.** Transfer status was ascertained objectively from contemporaneous medical and transport records and defined identically for both groups; only the secondary reason-for-transfer categorisation relied on an assumption (medical reason assumed when not documented), which does not affect the transferred/non-transferred classification itself.
- **D4 Deviations from intervention: Moderate.** As a naturally occurring exposure there was no protocol to deviate from, but transferred patients systematically received more intensive co-interventions (NIV, invasive ventilation, ECMO) that are only partly inherent to the transfer pathway and could influence outcomes.
- **D5 Missing data: Moderate.** In-hospital mortality was essentially complete (759/760), but 14 patients with unknown external admission dates (plausibly transfer-in patients) were excluded from the length-of-stay analysis and several baseline covariates had substantial missingness (BMI n=360, living situation n=486, APACHE-II n=303).
- **D6 Measurement of outcome: Low.** In-hospital death and length of stay are objective outcomes taken uniformly from hospital records for both groups; the main caveat is that in-hospital-only ascertainment truncates follow-up at discharge or transfer to non-acute/external units and misses post-discharge deaths (differential for transferred-out patients).
- **D7 Selection of reported result: Moderate.** No pre-registered protocol is cited, numerous subgroup analyses were reported with p-values explicitly not corrected for multiplicity, and multivariable adjustment was performed for the length-of-stay endpoint but not for the mortality endpoint.

**Toss Agegard 2025 — Overall: Serious**

- **D1 Confounding: Serious.** Strong confounding by indication in the opposite direction to most transfer studies: transfer decisions selected patients who were respiratory/haemodynamically stable with recovery potential and fewer treatment limitations, and although models adjusted for age, sex, BMI, SAPS III, CCI, invasive ventilation, treatment limitations and pandemic wave, severity immediately before transport was unavailable and the authors explicitly concede residual confounding, plausibly driving the apparent protective HR.
- **D2 Selection of participants: Serious.** Immortal time bias: exposure (transfer) occurred at a median of day 6 after ICU admission but follow-up in the main model started at ICU admission, so transferred patients were guaranteed to survive to transfer while early deaths fell into the non-transferred group; this was only partially mitigated by LOS-stratified sensitivity analyses (>6/8/10/12 days) and inflow-transferred patients (n=5) were excluded.
- **D3 Classification of intervention: Low.** Exposure (capacity-driven IHT) was an objective, well-defined event ascertained from medical records and the patient data management system (date, time and reason of transfer), classified independently of and prior to outcome ascertainment.
- **D4 Deviations from intervention: Low.** Transfers were carried out per Swedish SFAI transport guidelines via staffed mobile ICU and the exposure of interest (the transfer itself) was implemented as intended; no meaningful group-differential co-intervention beyond the exposure was identified, aside from intubation to secure transport which is intrinsic to the exposure.
- **D5 Missing data: Moderate.** The key severity confounder SAPS III was missing for 105 patients (~16%) and BMI for 6, handled with a missing-indicator category (a suboptimal method that can bias adjustment); outcome data were complete and a complete-case sensitivity analysis was unchanged, limiting but not eliminating concern.
- **D6 Measurement of outcome: Low.** Mortality at 30 and 90 days is an objective, hard endpoint obtained from records/registry, ascertained identically for both groups and not influenced by knowledge of exposure status.
- **D7 Selection of reported result: Low.** Analysis followed a predefined protocol with STROBE reporting; both crude and adjusted estimates for both timepoints plus multiple prespecified sensitivity/subgroup analyses (LOS strata, waves, ventilation, treatment limitations) were reported, giving little indication of selective result reporting.

**Wortel 2022 — Overall: Moderate**

- **D1 Confounding: Moderate.** Confounders were selected via a Directed Acyclic Graph and the Cox model adjusted for age, APACHE IV mortality probability, BMI, mechanical ventilation and vasoactive medication, but the authors acknowledge residual confounding because intensivists select transfer candidates on information not captured in the registry and capacity- vs clinical-driven transfers could not be distinguished (confounding by indication).
- **D2 Selection of participants: Low.** Immortal time bias (transferred patients must survive to transfer) was explicitly and appropriately handled by left-truncation at the day of transfer and confirmed by sensitivity analyses excluding patients discharged within 1 and 3 days.
- **D3 Classification of intervention: Low.** Transfer exposure was defined by an objective registry algorithm (continuation of ICU care in a different hospital's ICU, with <3-day inter-ICU gaps counted as transfers) derived from admission/discharge dates independent of the outcome.
- **D4 Deviations from intervention: Low.** As an observational registry study, exposure was analyzed as observed; grouping of the 3.8% transferred more than once and the 46 cross-border transfers together is a minor simplification unlikely to bias the effect.
- **D5 Missing data: Low.** Outcome (death) was near-completely ascertained through the national NICE registry linked to the Vektis claims database (~97% population coverage); patients with missing core clinical data or mortality status were excluded, but the excluded fraction was modest and outcome data essentially complete.
- **D6 Measurement of outcome: Low.** 180-day and in-hospital mortality are objective outcomes ascertained identically for both groups from registry and national death-date records, with no possibility of assessor influence by exposure status.
- **D7 Selection of reported result: Low.** The DAG-specified adjusted 180-day mortality analysis was reported, and the unfavorable post-hoc time-split result (HR 1.67 after 30 days) was transparently disclosed, though the absence of a cited pre-registered protocol leaves minor uncertainty.
