# Supplementary material for: Inter-hospital ICU-to-ICU transfer of critically ill COVID-19 patients is not associated with increased mortality: a systematic review with exploratory meta-analysis
Source: Sci Rep. 2026 Aug 8;16:24551. doi: 10.1038/s41598-026-62965-7 (PMC13452817; doi:10.1038/s41598-026-62965-7)
Supplement: Supplementary file 6 [file 41598_2026_62965_MOESM7_ESM.docx]

**Additional file 2. Full-text exclusions (n = 52)**

Reports excluded at the full-text stage with reasons. The final four entries were excluded after detailed full-text data assessment and are counted as full-text exclusions, consistent with the PRISMA flow (Figure 1). Exclusion codes: A1 no non-transferred comparator; A2 intra-hospital transfer; A3 not ICU-to-ICU; A4 no comparative clinical outcomes; A5 ECMO-specific transfer programme; A6 population not ICU-specific; A7 not original research.

| **PMID** | **First author** | **Year** | **Title** | **Code** | **Reason** |
| --- | --- | --- | --- | --- | --- |
| 33233987 | Salas de Armas | 2021 | Inter-hospital COVID ECMO air transportation. | A1 | No non-transferred comparator; ECMO transport case series (n=14) |
| 34172226 | Schaalma | 2021 | Analysis of European Air Medical Evacuation Flights of COVID-19 Patients. | A1 | No non-transferred comparator; military air evacuation descriptive analysis |
| 39319807 | Gonzalez | 2024 | Interfacility Patient Transfers During COVID-19 Pandemic: Mixed-Methods Study. | A4 | Transfer coordination system evaluation; no T-vs-NT clinical outcome comparison |
| 35950299 | Bos | 2022 | Managing Hospital Capacity: Achievements and Lessons from the COVID-19 Pandemic. | A4 | Hospital capacity management; no T-vs-NT outcome comparison |
| 34675022 | Oudar | 2021 | TRANSCOV cohort protocol: epidemiological study assessing impact of COVID-19 patient long distance transfers between ICUs. | A7 | Study protocol only; no results reported |
| 35226964 | Sherren | 2022 | Patient outcomes following transfer between intensive care units during the COVID-19 pandemic. | A1 | No non-transferred comparator; compares inter- vs intra-hospital ICU transfer routes |
| 33232835 | Boutonnet | 2021 | Collective aeromedical transport of COVID-19 critically ill patients in Europe: A retrospective study. | A1 | No non-transferred comparator; collective aeromedical transport cohort (n=36) |
| 39236274 | Schultz | 2024 | Policy Proposals for Mitigating Intensive Care Unit Strain: Insights from the COVID-19 Pandemic. | A7 | Narrative review; no original patient data |
| 33228902 | Singh | 2021 | Interhospital Transfer of Critically Ill Patients Because of COVID-19-Related Respiratory Failure. | A4 | Transport team safety and logistics; no T-vs-NT clinical outcome comparison |
| 40188194 | Garcia-Salmones | 2025 | A transfer triage tool for COVID-19 mass critical care surges. | A1 | No non-transferred comparator; triage tool development for transferred patients only |
| 37225747 | Pietsch | 2023 | Burden of hospital admissions and resulting patient interhospital transports during SARS-CoV-2 pandemic in Saxony. | A4 | Transport epidemiology; COVID vs non-infectious transports; no T-vs-NT comparison |
| 40947944 | Shrestha | 2025 | Challenges and implementation of ECMO transport program in a low-resource setting. | A5 | ECMO-specific transport program; no general ICU-to-ICU T-vs-NT comparison |
| 39313793 | Hattenhauer | 2024 | Assessing the impact of Delta and Omicron in German ICUs: a retrospective nationwide multistate analysis. | A4 | Variant comparison (Delta vs Omicron); inter-hospital transfer not primary exposure |
| 34130948 | Longhini | 2021 | Risk factors for transfer from Respiratory Intermediate Care Unit to Intensive Care Unit in COVID-19. | A2 | Intra-hospital transfer (RICU to ICU); not inter-hospital |
| 34088335 | Lorusso | 2021 | Collective Critical Care Ambulance: innovative transportation of critical care patients by bus in COVID-19. | A4 | Transport logistics (bus adaptation); no T-vs-NT clinical outcome comparison |
| 35049190 | Baudin | 2022 | Emergency air evacuation of patients with acute respiratory failure due to SARS-CoV-2 from Mayotte to Reunion Island. | A1 | No non-transferred comparator; air evacuation case series |
| 38816850 | Peeters | 2024 | COVID-19 in three waves in a tertiary referral hospital in Belgium: comparison of patient characteristics and outcome. | A4 | Wave comparison at single center; inter-hospital transfer not primary exposure |
| 39617740 | Mabasa | 2024 | Establishing an interhospital ECMO-transport program during a pandemic: Key lessons learned. | A5 | ECMO-specific transport program; no general ICU T-vs-NT comparison |
| 33647975 | Sef | 2021 | Initiation of inter-hospital ECMO transfer programme for critically ill COVID-19 patients. | A5 | ECMO-specific transfer program; no non-transferred comparator |
| 33297986 | Ciardullo | 2021 | Clinical characteristics and laboratory biomarkers changes in COVID-19 patients requiring or not intensive or sub-intensive care. | A2 | Intra-hospital triage (ward to ICU/SICU); not inter-hospital transfer |
| 35661838 | Hughes | 2022 | Role of retrieval nurse practitioners in safely transferring critically ill adults and children during COVID-19. | A4 | Transport nursing practice; no clinical outcome comparison |
| 37254008 | Elmer | 2023 | Inter-hospital Transfer Decision-making During the COVID-19 Pandemic: a Qualitative Study. | A7 | Qualitative study; no quantitative clinical outcomes |
| 36123727 | van den Berg | 2022 | Safety during interhospital helicopter transfer of ventilated COVID-19 patients. | A4 | Transport physiology (vital signs during flight); no T-vs-NT mortality comparison |
| 34026050 | Sangwan | 2021 | Post-COVID recovery: chronically critically ill patients admitted to a long-term acute care hospital. | A3 | Transfer to LTACH (not ICU-to-ICU) |
| 32764134 | Pasquier | 2020 | Critical COVID-19 patient evacuation on an amphibious assault ship: feasibility and safety. | A1 | No non-transferred comparator; military evacuation case series (n=12) |
| 37146598 | Guenther | 2023 | Transfer of VV-ECMO Patients With COVID-19 Associated ARDS. | A5 | ECMO-specific; compares mobile ECMO transfer vs in-center ECMO cannulation |
| 37313688 | Garibaldi | 2023 | The Maryland Critical Care Coordination Center (C4): From Pandemic to Permanence. | A4 | Coordination center description; no T-vs-NT clinical outcome comparison |
| 41324958 | Mohr | 2025 | Statewide Transfer Coordination and Patient Transfer Rates Among Hospitals During Occupancy Stress. | A4 | Transfer coordination policy evaluation; no T-vs-NT clinical outcomes |
| 36901672 | Roux | 2023 | Experiences of Inter-Hospital Transfers by Patients and Relatives during COVID-19 in France: A Qualitative Study. | A7 | Qualitative study; patient and relative experiences; no clinical outcomes |
| 35750448 | Contreras | 2022 | SARS-CoV-2 Pandemic: Chilean Air Force Experience in Air Transport of Critically Ill Patients. | A1 | No non-transferred comparator; military air transport case series (n=100) |
| 35289365 | Berard | 2022 | Effect of a prioritization score on inter-hospital transfer time management of severe COVID-19. | A4 | Transfer prioritization/triage tool; no T-vs-NT outcome comparison |
| 37667885 | Cabrini | 2023 | SARS-CoV-2 and Medical Evacuation in Lombardy: Lessons Learned from an Unprecedented Pandemic. | A1 | No non-transferred comparator; medical evacuation description (n=119) |
| 35103441 | Bello | 2022 | Priority in interhospital transfers of patients with severe COVID-19: development and prospective validation of a triage tool. | A4 | Triage tool development and validation; no T-vs-NT outcome comparison |
| 34176676 | Gannon | 2021 | Implementation and Outcomes of a Mobile ECMO Program in the United States During the COVID-19 Pandemic. | A5 | ECMO-specific program; no general ICU T-vs-NT comparison |
| 34802012 | Sromicki | 2021 | Mobile ECMO for Covid-19 Does Not Pose Extra Risk to Transport Team. | A4 | Transport team safety analysis; no clinical outcome comparison |
| 38185073 | Lefaucheur | 2024 | Psychological impact of medical evacuation for ICU saturation in Covid-19-related ARDS patients. | A4 | Psychological outcomes only (PTSD); not mortality or ICU-LOS |
| 33627596 | Badulak | 2021 | Extracorporeal Membrane Oxygenation for COVID-19: Crisis Standards of Care. | A7 | Review and guidance document; no original T-vs-NT comparison |
| 37093031 | Matsumoto | 2023 | Managing Interhospital Referrals During a COVID-19 Patient Surge in Japan. | A4 | Referral management and bed exchange logistics; no T-vs-NT clinical outcomes |
| 37357595 | Powell | 2023 | Timing of Transport of Patients with COVID-19. | A1 | No non-transferred comparator; all patients transferred; timing analysis only |
| 35317693 | Ramzy | 2022 | Pushing Geographic Boundaries: Interfacility transport and remote ECMO cannulation during COVID-19. | A5 | ECMO-specific interfacility transport; no general ICU T-vs-NT comparison |
| 36464607 | Evrard | 2022 | One hundred ECMO retrievals before and during the Covid-19 pandemic: an observational study. | A5 | ECMO-specific retrieval program; COVID vs non-COVID comparison |
| 41528394 | Feisst | 2025 | Experiences from SARS-CoV-2 pandemic at UKSH Luebeck. | A1 | Descriptive single-center experiences; no adequate non-transferred ICU comparator |
| 32885172 | Cummings | 2020 | Clinical Outcomes in Critically Ill COVID-19 Patients: A Unique New York City Public Hospital Experience. | A1 | Transfer variable incidental; no T-vs-NT comparison as primary analysis |
| 35403027 | Salna | 2022 | Implementation and outcomes of an urban mobile adult extracorporeal life support program. | A5 | ECMO/ECLS-specific program; no general ICU T-vs-NT comparison |
| 39336878 | Langguth | 2024 | Fit-for-Future: Lessons Learned from COVID-19 in Primary ECMO Transports of ARDS Patients. | A5 | ECMO-specific; pre-pandemic vs pandemic comparison; no T-vs-NT |
| 37693304 | Calero | 2023 | Single-Center Experience With Lung Transplant Evaluation Referrals of ARDS Patients During COVID-19. | A3 | Lung transplant referral pathway; not general ICU-to-ICU transfer |
| 37492857 | Siow | 2023 | Outcomes of Patients Transported in the Prone Position to a Regional ECMO Center. | A5 | ECMO-specific (prone transport to ECMO center); no general ICU T-vs-NT comparison |
| 34830505 | Pasquier | 2022 | Impact of Air Transport on SpO2/FiO2 among Critical COVID-19 Patients during First Pandemic Wave in France. | A4 | Transport physiology (SpO2/FiO2 changes); no T-vs-NT mortality comparison |
| 33745381 | Frakes | 2021 | Critical Care Transport of Patients With COVID-19. | A1 | No non-transferred comparator (single-arm transport cohort); excluded after full-text data assessment |
| 40009524 | Fagoni | 2025 | The leading 'blended' civil medical evacuation operation (MEDEVAC) in unforeseen health emergency. | A1 | No comparative non-transferred control cohort (MEDEVAC logistics); excluded after full-text data assessment |
| 39690357 | Pestana | 2025 | The burden of COVID-19 care in community and academic intensive care units in Ontario, Canada. | A1 | No direct transfer-versus-non-transfer comparison (compares hospital types); excluded after full-text data assessment |
| 34569998 | Usher | 2022 | Responding to COVID-19 Through Interhospital Resource Coordination: A Mixed-Methods Evaluation. | A6 | Population not ICU-specific (~32% in ICU); excluded after full-text data assessment |
