# Supplementary material for: Inter-hospital ICU-to-ICU transfer of critically ill COVID-19 patients is not associated with increased mortality: a systematic review with exploratory meta-analysis
Source: Sci Rep. 2026 Aug 8;16:24551. doi: 10.1038/s41598-026-62965-7 (PMC13452817; doi:10.1038/s41598-026-62965-7)
Supplement: Supplementary file 7 [file 41598_2026_62965_MOESM8_ESM.docx]

**Additional file 8. Expanded study characteristics, estimands, and outcome definitions**

**Table S3. Study designs, populations, transfer definitions, and target estimands**

| **Study (country; design)** | **Setting & period** | **Inclusion / exclusion** | **Primary outcome** | **Transfer definition & direction** | **Effect measure** | **Target estimand** |
| --- | --- | --- | --- | --- | --- | --- |
| Chen 2021 (USA) | 1 (single receiving tertiary referral center — Rush University Medical Center; transfers came from multiple unnamed community/acute-care hospitals, but analysis is single-center at the receiving site). centre(s); Retrospective analysis of the electronic... | IN: Adults admitted to RUMC with a positive SARS-CoV-2 test during hospitalization or in the week prior to admission, with COVID-19 pneumonia, who were admitted to an ICU AND received invasive mechanical... EX: No explicit exclusion list. Implicit exclusions from the inclusion funnel (Fig. 1): patients not admitted to an ICU and patients not intubated (did not receive... | Hospital mortality (including discharge to hospice) versus survival to hospital discharge. Secondary outcome: number of days from initial intubation (in-institution) to hospital discharge or death,... | Transfer IN only. Interhospital transfer INTO the tertiary referral center: patients "transferred from another acute care hospital" versus patients admitted through the RUMC ED. Capacity/surge-driven and clinically driven — the center... | Adjusted OR 1.45 (0.86-2.43) | Effect of being transferred INTO a tertiary centre among mechanically ventilated ICU patients; transferred group is transport-stable and survived to acceptance (survivor selection). |
| Cini 2023 (Australia) | 63 participating ICUs. centre(s); Registry: SPRINT-SARI Australia (Short Period Incidence Study of Severe Acute Respiratory Infection), a national prospective observational ICU cohort. Data collected from both electronic and paper medical records, entered... | IN: Consecutive patients with polymerase chain reaction (PCR)-confirmed SARS-CoV-2 infection admitted to one of the 63 participating ICUs that contributed data during 1 January 2020 – 1 April 2022.... EX: No explicit exclusion criteria stated (NR). Analyses omitted missing data variable-by-variable ("summarised as counts and proportions (omitting missing... | In-hospital mortality (stated primary outcome). Secondary outcomes: ICU and hospital lengths of stay, and frequency of selected complications. | Direction unclear. Mixed capacity-driven and clinical indication. Transfers occurred "typically to provide access to higher level care, because clinical demand was unevenly distributed, or particular hospitals were under strain." Exposure... | Adjusted RD -5.0 pp (-10 to -0.03) | Effect of ANY transfer (including transfer for higher-level therapies such as ECMO, 26% vs 2%); transfer partly marks illness severity, not only capacity. |
| Grimaud 2026 (France) | Multicenter. Of 70 origin hospitals and 146 destination hospitals nationally, 46/70 origin hospitals (66%) and 65/146 destination hospitals (45%) took part in the study. centre(s); Medical records: data extracted from ICU health records at origin hospitals... | IN: Transferred: all ICU patients with severe COVID-19 transferred inter-regionally (ICU-to-ICU, in France or abroad) between 13 March and 10 April 2020, with complete information available on both... EX: Transferred patients lacking complete origin+destination ICU data (174 of 502 enrolled) and those without a matchable control; controls who died or were... | 28-day ICU case fatality (28-day mortality in intensive care after transfer) of transferred vs matched non-transferred patients, expressed as an incidence rate ratio (IRR) from mixed Poisson models. | Transfer OUT only. Capacity-driven mass medical evacuation (not ECMO/clinical): during a first-wave surge with ICU bed occupancy >200%, severe COVID-19 patients were transferred ICU-to-ICU out of overwhelmed origin ICUs to destination... | PS-weighted IRR 0.14 (0.10-0.19) | Effect of capacity-driven mass evacuation among patients surviving to 5 days post-transfer (day-5 conditioning); estimand conditions on post-transfer survival. |
| Painvin 2021 (France) | 3 (three teaching/university hospital ICUs: Rennes n=47 [35%], Tours n=43 [32%], Poitiers n=43 [32%]). centre(s); Retrospective analysis of medical records ("Data were retrospectively analysed from medical records in 3 teaching hospitals in the West of France... | IN: All patients >18 years admitted to the 3 study ICUs (Poitiers, Rennes, Tours) between 15 March and 15 April 2020 requiring invasive mechanical ventilation for COVID-19 confirmed by... EX: No explicit exclusion criteria stated beyond the inverse of the inclusion criteria (implicitly: age <18 years, no invasive mechanical ventilation, and... | ICU mortality. Stated main objective: "The main objective was to compare the ICU mortality between patients who underwent inter-hospital transfer during their ICU stay and patients who did not."... | Transfer IN only. Capacity-/surge-driven inter-hospital evacuation (not ECMO- or clinical-deterioration-driven): critically ill mechanically ventilated COVID-19 patients were transferred from overcrowded ICUs (East of France and Paris... | Adjusted OR 1.20 (0.35-4.09) | Effect of capacity-driven transfer IN across three centres; small sample; unadjusted for baseline severity. |
| Sanchez 2021 (France) | National — all French hospitals reporting to the SI-VIC registry (every hospital in France recorded COVID-19 cases daily). Exact number of participating centres/ICUs not reported (NR). centre(s); National SI-VIC online health emergency registry (French... | IN: All patients registered in SI-VIC and admitted to a French ICU for COVID-19 between 1 March and 21 June 2020; COVID-19 biologically confirmed by positive RT-PCR or highly suspected on chest CT.... EX: Complete-case analysis: excluded ICU admissions with incomplete data (n=817); lost to follow-up during ICU stay (n=328) or during post-ICU acute care (n=265)... | In-hospital mortality (stated as "in-hospital mortality" in the abstract and "in-hospital acute care mortality" in the Methods). | Transfer OUT only. Capacity/surge-driven inter-hospital ICU-to-ICU transfer from overwhelmed toward less-saturated ICUs (intra-regional, or inter-regional/international). Triggered at the local/intrahospital level (validated by regional... | Distance-stratified RR 0.38-0.98 | Effect of transfer OUT during ICU care in the national cohort; capacity-driven; distance-stratified. |
| Stark 2023 (UK) | Single sending (index) centre — University Hospital Monklands — with 7 receiving ICUs within the WoSCCN. Effectively single-centre recruitment (all patients admitted to one unit). centre(s); Registry / clinical audit database: SICSAG (Scottish Intensive Care... | IN: All patients admitted to the University Hospital Monklands ICU between 1 October 2020 and 31 March 2021 with a primary diagnosis of COVID-19 pneumonia. EX: Interhospital transfers for reasons other than capacity (i.e., not for bed or staff shortage) were excluded. Readmission episodes were excluded from the... | Survival to ultimate hospital discharge (analysed by backward stepwise logistic regression, transfer status forced in; reported as adjusted OR). Secondary outcomes: total ventilator days and total... | Transfer OUT only. Capacity-driven interhospital ICU-to-ICU transfer, defined as transfer OUT of the base hospital due to unit shortage of beds or unit shortage of staff; transfers for any other (e.g., clinical/specialist) reason were... | OR for survival 1.14 (0.43-3.1); inverted OR mortality 0.88 | Effect of capacity transfer OUT from a district general hospital; transferred patients must be fit for transfer. |
| Suski 2024 (Germany) | 4 (RoMed acute-care hospitals: Rosenheim [Level 2, 622 beds], Wasserburg [Level 1], Bad Aibling [Level 1], Prien am Chiemsee [Level 1]); transfers additionally involved hospitals external to the RoMed group. centre(s); Retrospective analysis of anonymized... | IN: All patients admitted 1 Aug-31 Dec 2021 to one of the four RoMed acute-care hospitals (as primary admission or after transfer from an external clinic), with a positive SARS-CoV-2 PCR (Ct <=30) on... EX: Positive PCR not clinically linked to the admission (e.g., obstetric/trauma patients with an incidental positive test and no COVID-19 symptoms) - 52 of 812... | The study's stated major/primary outcome was the total period of hospitalization (length of stay); in-hospital mortality was the co-primary clinical outcome, together with identification of factors... | Transfer in & out. Any inter-hospital transfer during the COVID-19 hospitalization in which one of the four RoMed network hospitals was involved: either transfer between the four network hospitals, or transfer to/from a hospital external... | No adjusted effect estimate (p=0.663) | Effect of transfer (mixed capacity/medical/ECMO reasons); ICU stratum used; no adjusted mortality estimate. |
| Toss Agegard 2025 (Sweden) | Single index centre (Sodersjukhuset), comprising two ICUs (single-centre cohort study). Transferred patients were sent OUT to other, unspecified receiving hospitals within the Stockholm region. centre(s); Medical records and patient data management systems... | IN: All adult patients (age >=18 years) with PCR-confirmed SARS-CoV-2 requiring intensive care whose INITIAL ICU admission was at the study hospital (Sodersjukhuset, Stockholm), between 1 March 2020 and... EX: Patients first admitted to other ICUs and subsequently transferred INTO the study hospital (n=5); patients transported for medical reasons such as ECMO (n=9);... | No single primary outcome is designated. Stated main outcome measures: 30-day and 90-day mortality (from ICU admission) and days alive and out of ICU. HRs were the headline effect estimates (adjusted... | Transfer OUT only. Capacity-driven only. IHT defined as "the transfer of patients from the ICU at the index hospital to another hospital's ICU due to capacity constraints" (shortage of staffed ICU beds at the referring hospital).... | Adjusted HR 0.47 (0.30-0.76), 30-day | Left-truncated design: effect of transfer OUT among patients well enough (and surviving) to be transferred - a 'less unwell' selected cohort. |
| Wortel 2022 (Netherlands) | 73 ICUs in 68 hospitals (all Dutch hospitals that treated COVID-19 ICU patients). centre(s); National registry: Dutch National Intensive Care Evaluation (NICE) registry, a quality registry covering all Dutch ICUs (minimal dataset + a dedicated COVID-19... | IN: Adult COVID-19 patients admitted to Dutch ICUs between 1 March 2020 and 1 July 2021 with either a positive SARS-CoV-2 RT-PCR (nasopharyngeal swab) or a CT scan consistent with COVID-19 (CO-RADS score... EX: ICU length of stay <4 h; missing core clinical MDS data; admission type elective or emergency surgery; medical admission type with a non-COVID-19-related... | The study did not formally label a single "primary outcome," but the principal analyzed outcome for the transferred-vs-non-transferred comparison was all-cause mortality during the first 180 days... | Transfer in & out. A transfer was defined as continuation of ICU treatment in an ICU of a different hospital (inter-hospital ICU-to-ICU). Ward-to-ICU or ICU-to-ward moves between hospitals were NOT counted as transfers. Gaps of <3 days... | Adjusted HR 0.99 (0.91-1.08), 180-day | Left-truncated national cohort: effect of transfer among patients surviving to transfer; strongest severity adjustment (APACHE IV mortality probability). |

**Table S4. Exact mortality definition, timepoint, and length-of-stay composition per study**

| **Study** | **Mortality definition & timepoint** | **ICU LOS - what is measured** | **Hospital LOS - what is measured** |
| --- | --- | --- | --- |
| Chen 2021 | In-hospital mortality (death prior to hospital discharge), with the study's primary outcome defined as hospital mortality INCLUDING discharge to hospice vs survival to discharge; no fixed day timepoint. | ICU days = receiving-center (RUMC) only. Median ICU days: transfers 14 (IQR 6-27) vs ED admits 18 (IQR 9-25), p=0.069 (Table 2). The authors explicitly could not access hospital-of-origin data, so pre-transfer ICU time at the sending hospital is NOT included; for transfers the counter effectively starts at RUMC ICU admission. Not a total cross-hospital ICU episode. | Reported as "time (in days) from first ventilation day (D0) to discharge" = receiving-center (RUMC) only: transfers 17 (IQR 8-31) vs ED admits 20 (IQR 9-28), p=0.918 (Table 2). Anchored at D0 (first invasive ventilation day for ED admits; ICU admission date for transfers already ventilated on arrival). Pre-transfer/sending-hospital length of stay is not included; not a total... |
| Cini 2023 | In-hospital (all-cause) mortality during the index hospitalisation — the study's stated primary outcome. Crude/complete-case comparison of deaths in hospital between transferred and non-transferred groups (no fixed day cut-off; hospital episode-based). Survival curves depicted to day 90 from hospital admission. | Total ICU episode across both sending and receiving hospitals — for transferred patients ICU LOS was explicitly "calculated as the total number of days in intensive care since the index admission to hospital" (i.e., cumulative days in intensive care from the first/index hospital admission, spanning the referring and receiving centres). Transferred median ICU LOS 20.0 days (IQR... | Total hospital episode across both sending and receiving hospitals, anchored at the index (first) hospital admission. Consistent with the ICU-LOS convention, hospital LOS captures the whole cross-hospital journey; Kaplan–Meier survival and disposition figures are indexed to "time since [hospital] admission" (Box 4: "Survival to day 90 from hospital admission"; Box 5:... |
| Grimaud 2026 | 28-day ICU case fatality (in-ICU death within 28 days), with follow-up anchored at 5 days after the transfer date; this is the study's main transferred-vs-control comparison. | Total ICU episode aggregated across both sending (origin) and receiving (destination) ICUs for transferred patients; for controls it is the origin-ICU stay only. Table 3 footnote: \"The whole ICU stay includes both origin and destination ICU stays for transferred patients.\" Reported as median days: transferred 23 (IQR 16-35) vs control 21 (14-34), P=.004. Start point (ICU... | NR — hospital length of stay is not reported; only ICU length of stay is given. |
| Painvin 2021 | ICU mortality (death during the ICU stay) — the study's stated main/primary comparison. Crude counts reported; the adjusted analysis gave an odds ratio of 1.2 (95% CI 0.35-4.09, p=0.76) after adjustment for AKI, number of proning sessions, and duration of mechanical ventilation. Hospital mortality also reported separately (8 [12%] vs 8 [12%], p=0.92). | ICU length of stay: median 22 days (IQR 16-32) transferred vs 19 (11-28) non-transferred, p=0.07. The Table 1 sub-heading ("outcome and support ... over stay in Poitiers, Rennes, and Tours ICUs") suggests these are measured at the receiving centres, and the pre-transfer ICU stay (median 7.5 days, IQR 4-11) is reported separately — implying ICU LOS likely reflects the... | Hospital length of stay: median 36 days (IQR 24-48) transferred vs 30 (19-45) non-transferred, p=0.11. The paper does not state whether hospital LOS includes the sending-hospital stay prior to transfer; consistent with the receiving-centre chart-based data collection it likely reflects the receiving-hospital stay only, but this is not explicit. Anchor: unclear, probably... |
| Sanchez 2021 | In-hospital mortality (in-hospital acute care mortality); no fixed day cut-off — follow-up to 14 July 2020. This is the study's pre-specified primary endpoint for the transferred vs non-transferred comparison. | ICU length of stay reported as "Length of stay in ICU, days (mean, SD)": non-transferred 13.5 (14.6), intra-regional transfer 30.6 (21.8), inter-regional/international 29.9 (19.3). Because all hospital stays are linked via a unique national identifier and the registry separately records "LOS in ICU before transfer" and total "LOS in ICU", the ICU LOS most plausibly represents... | Hospital LOS reported as "Total length of stay in acute care, days (mean, SD)": non-transferred 19.8 (17.0), intra-regional 36.9 (22.7), inter-regional/international 38.0 (20.1). Represents the total acute-care hospital course (acute unit + ICU) across both sending and receiving facilities via national-identifier linkage; start point = acute-care/hospital admission. Acute-care... |
| Stark 2023 | In-hospital mortality — death before ultimate hospital discharge (the inverse of the study's primary outcome 'survival to ultimate hospital discharge'; all patients followed to completion of their hospital stay). No fixed day cut-off (not 28/30/90-day). | "Total intensive care unit length of stay (Calendar days)" — median 7.5 (IQR 3.3-15) for non-transfers vs 25 (IQR 16-41) for transfers (Table 2). Start point = ICU admission at the base hospital. Data drawn from the base hospital's SICSAG WardWatcher audit database; the paper labels it 'total' ICU LOS but does not state whether ICU time accrued at the receiving unit after... | NR — hospital length of stay is not reported as a numeric outcome. Only survival to ultimate hospital discharge (primary outcome) and ICU LOS / ventilator days are reported. (Patients were followed to completion of their hospital stay, but no hospital-LOS days are given.) |
| Suski 2024 | In-hospital (all-cause) mortality during the acute-care hospital episode; the main transferred-vs-non-transferred comparison is reported separately for ICU and non-ICU patients (ICU stratum used here as the ICU-to-ICU-relevant comparison). Post-discharge mortality was not captured. | 'Stay on ICU in a RoMed hospital' = ICU time within the RoMed network only (receiving/index-network ICU time), NOT the total ICU episode across sending + receiving hospitals; ICU time spent at external hospitals is not counted. Reported for ICU patients: non-transferred median 6.0 (3.0-9.0) vs transferred 10.0 (6.0-20.0) days, p<0.001 (n=208). | 'Hospital stay, total time (days)' = total period of hospitalization summed across ALL involved hospitals (sending + receiving), starting at the date of first admission to a RoMed hospital, or at admission to the external clinic when the patient was primarily admitted externally and that date was known; end = release from an acute-care hospital or transfer to a non-acute unit... |
| Toss Agegard 2025 | 30-day all-cause mortality from date of ICU admission (co-primary with 90-day mortality). Counts below are the 30-day figures; corresponding 90-day counts: IHT 34/133 (26%), non-IHT 157/532 (30%). Main highlighted adjusted estimate: 30-day mortality HR 0.47 (95% CI 0.30-0.76); 90-day HR 0.52 (95% CI 0.34-0.78/0.79). | Total ICU episode spanning both sending and receiving hospitals. Verbatim: "The length of ICU stay was measured from the date of ICU admission to the date of discharge from the ICU at Sodersjukhuset or the admitting hospital." Start point = initial ICU admission at the index hospital; endpoint = final ICU discharge (at index or receiving hospital). Note: the study reports... | NR. Although the title/abstract mention "length of hospital stay," no hospital LOS (median/IQR) is actually reported; the analysed length-related outcome is ICU-based (days alive and out of ICU / ICU LOS anchor). Hospital LOS not extractable. |
| Wortel 2022 | All-cause mortality during the first 180 days after (initial) ICU admission (180-day mortality). This was the outcome used in the main transferred-vs-non-transferred comparison via a left-truncated (truncation at day of transfer) multivariable Cox model; adjusted HR 0.99 (95% CI 0.91-1.08). | Total ICU length of stay across all ICU episodes (summed over multiple admission periods), starting from first ICU admission; for transferred patients this spans both the sending and receiving ICU. Reported as "Total ICU length of stay" median (IQR): non-transferred 10.0 (5.0-19.0) days vs transferred 18.0 (11.0-32.0) days. The paper also separately reports transferred... | Total hospital length of stay across the entire admission trajectory (the registry explicitly calculated total length of stay over multiple admission periods), i.e., for transferred patients spanning both sending and receiving hospitals, anchored from initial admission. Median (IQR): non-transferred 18.0 (11.0-31.0) days vs transferred 26.0 (17.0-42.0) days (Table 1). |

*Estimand heterogeneity: the included studies target different parameters, ranging from the effect of inter-ICU transfer at any time point (e.g., Cini, where transfer may in part mark illness severity) to the effect of transfer among patients well enough to be transferred (e.g., Toss Agegard and Wortel, whose left-truncated designs condition on survival to transfer). Aggregate data are not granular enough to quantify the proportions of capacity-driven versus clinically driven (e.g., ECMO) transfers within each cohort.*
