# Supplementary material for: Inter-hospital ICU-to-ICU transfer of critically ill COVID-19 patients is not associated with increased mortality: a systematic review with exploratory meta-analysis
Source: Sci Rep. 2026 Aug 8;16:24551. doi: 10.1038/s41598-026-62965-7 (PMC13452817; doi:10.1038/s41598-026-62965-7)
Supplement: Supplementary file 8 [file 41598_2026_62965_MOESM9_ESM.docx]

**Additional file 9. Crude-effect sensitivity meta-analysis (odds ratios, Hartung-Knapp)**

As a sensitivity analysis addressing the differing effect measures across studies, crude odds ratios for mortality were derived from 2×2 counts extracted from each report (verified against the source text) and pooled using random-effects models. Both DerSimonian-Laird (DL) and Hartung-Knapp-Sidik-Jonkman (HK) confidence intervals are reported. This crude pool is exploratory: the primary synthesis uses adjusted estimates, mortality timepoints differ across studies, and the pool mixes timepoints only to assess robustness of the direction of effect.

**Per-study crude odds ratios**

| **Study** | **Mortality timepoint** | **T deaths/N** | **NT deaths/N** | **Crude OR (95% CI)** |
| --- | --- | --- | --- | --- |
| Chen 2021 | in-hospital | 50/117 | 78/181 | 0.99 (0.62-1.58) |
| Cini 2023 | in-hospital | 60/309 | 827/4487 | 1.07 (0.80-1.43) |
| Grimaud 2026 | 28-day ICU (day-5 conditioned) | 20/285 | 199/667 | 0.18 (0.11-0.29) |
| Painvin 2021 | ICU | 7/68 | 8/65 | 0.82 (0.28-2.40) |
| Sanchez 2021 | in-hospital | 469/2228 | 4221/15303 | 0.70 (0.63-0.78) |
| Stark 2023 | in-hospital | 12/30 | 24/78 | 1.50 (0.63-3.60) |
| Suski 2024 | in-hospital | 39/114 | 31/100 | 1.16 (0.65-2.05) |
| Toss Agegard 2025 | 30-day | 25/133 | 136/532 | 0.67 (0.42-1.09) |
| Wortel 2022 | 180-day | 787/2814 | 2142/7395 | 0.95 (0.86-1.05) |

**Pooled random-effects estimates and sensitivity analyses**

| **Model** | **k** | **OR (DL 95% CI)** | **OR (HK 95% CI)** | **I² / τ²** | **95% PI** |
| --- | --- | --- | --- | --- | --- |
| Primary crude pool (excl. Grimaud) | 8 | 0.90 (0.74-1.08) | 0.90 (0.74-1.09) | 71% / 0.034 | 0.54-1.49 |
| Sensitivity: incl. Grimaud | 9 | 0.77 (0.59-1.00) | 0.77 (0.49-1.21) | 87% / 0.105 | 0.34-1.76 |
| Sensitivity: excl. unaddressed-ITB studies | 5 | 0.86 (0.70-1.06) | 0.86 (0.67-1.11) | 81% / 0.036 | 0.43-1.72 |
| Sensitivity: excl. French overlap extras | 7 | 0.90 (0.74-1.09) | 0.90 (0.73-1.12) | 75% / 0.036 | 0.52-1.56 |

**Forest plot (primary model, k=8)**

*DL, DerSimonian-Laird; HK, Hartung-Knapp-Sidik-Jonkman; PI, prediction interval; ITB, immortal time bias. Grimaud 2026 (day-5 conditioning) is an outlier; its inclusion shifts the pooled estimate downward and inflates heterogeneity. All confidence intervals cross the null.*
